# Comparison of Solution Chemical Properties and Biological Activity of Ruthenium Complexes of Selected *β*-Diketone, 8-Hydroxyquinoline and Pyrithione Ligands

**DOI:** 10.3390/ph14060518

**Published:** 2021-05-27

**Authors:** Tamás Pivarcsik, Gábor Tóth, Nikoletta Szemerédi, Anita Bogdanov, Gabriella Spengler, Jakob Kljun, Jerneja Kladnik, Iztok Turel, Éva A. Enyedy

**Affiliations:** 1 MTA-SZTE Lendület Functional Metal Complexes Research Group, University of Szeged, Dóm Tér 7, H-6720 Szeged, Hungary; pivarcsik.tamas@gmail.com (T.P.); gabor462600@gmail.com (G.T.); 2 Department of Inorganic and Analytical Chemistry, Interdisciplinary Excellence Centre, University of Szeged, Dóm Tér 7, H-6720 Szeged, Hungary; 3 Department of Medical Microbiology, Albert Szent-Györgyi Health Center, Faculty of Medicine, University of Szeged, Semmelweis u. 6, H-6725 Szeged, Hungary; szemeredi.nikoletta@med.u-szeged.hu (N.S.); varga-bogdanov.anita@med.u-szeged.hu (A.B.); 4 Faculty of Chemistry and Chemical Technology, University of Ljubljana, 1000 Ljubljana, Slovenia; jakob.kljun@fkkt.uni-lj.si (J.K.); jerneja.kladnik@fkkt.uni-lj.si (J.K.)

**Keywords:** MTT assay, UV-vis, solution stability, MRSA, albumin binding, ligand effect

## Abstract

In this work, the various biological activities of eight organoruthenium(II) complexes were evaluated to reveal correlations with their stability and reactivity in aqueous media. Complexes with general formula [Ru(η^6^-*p*-cymene)(X,Y)(Z)] were prepared, where (X,Y) represents either an *O*,*O*-ligand (*β*-diketone), *N*,*O*-ligand (8-hydroxyquinoline) or *O*,*S*-pyrithione-type ligands (pyrithione = 1-hydroxypyridine-2(1*H*)-thione) with Cl^−^ or 1,3,5-triaza-7-phosphaadamantane (PTA) as a co-ligand (Z). The tested complexes inhibit the chlamydial growth on HeLa cells, and one of the complexes inhibits the growth of the human herpes simplex virus-2. The chlorido complexes with *N*,*O*- and *O*,*S*-ligands displayed strong antibacterial activity on Gram-positive strains including the resistant *S. aureus* (MRSA) and were cytotoxic in adenocarcinoma cell lines. Effect of the structural variation on the biological properties and solution stability was clearly revealed. The decreased bioactivity of the *β*-diketone complexes can be related to their lower stability in solution. In contrast, the *O*,*S*-pyrithione-type complexes are highly stable in solution and the complexation prevents the oxidation of the *O*,*S*-ligands. Comparing the binding of PTA and the chlorido co-ligands, it can be concluded that PTA is generally more strongly coordinated to ruthenium, which at the same time decreased the reactivity of complexes with human serum albumin or 1-methylimidazole as well as diminished their bioactivity.

## 1. Introduction

Ruthenium complexes are prominent subjects in the development of chemotherapeutic agents, and some of them have entered clinical trials. Imidazolium *trans*-[tetrachlorido(dimethylsulfoxide)(1*H*-imidazole)ruthenate(III)] (NAMI-A) was the first Ru(III) complex introduced into such trials [1], while sodium *trans*-[tetrachloridobis(1*H*-indazole)ruthenate(III)] (NKP-1339) is at the moment one of the most investigated non-platinum drugs in clinical development [2]. A novel Ru(II) compound [Ru(4,4′-dimethyl-2,2′-bipyridine)_2_-(2-(2′,2″:5″,2‴-terthiophene)-imidazo-[4,5-f][1,10]-phenanthroline)]Cl_2_ (TLD-1433) has also entered a human clinical trial as a potential nontoxic photodynamic therapeutic agent [3]. Organoruthenium(II) complexes have also attracted great interest in chemotherapeutic studies and represent a versatile platform for the design of antitumor metal-containing drugs. Currently, the best-known prototypes of half-sandwich Ru(II) complexes are 1,3,5-triaza-7-phosphatricyclo-[3.3.1.1]decane (PTA) containing Ru(II)-arene compounds such as [Ru(η^6^-*p*-cymene) (PTA)Cl_2_] (RAPTA-C) with their significant antimetastatic properties being ready for translation into clinical evaluation [4] as well as the 1,2-ethylenediamine (en)-containing complexes such as [Ru(η^6^-biphenyl)(en)Cl]PF_6_ (RM175) [5,6].

The chemical properties and pharmacological activity of the Ru(II) half-sandwich complexes can be fine-tuned by varying the coordinated arene ring, the bidentate ligand (X,Y), and the co-ligand (Z) [4,7,8]. These components influence the size of the complex, its lipophilicity and charge as well as the strength of the coordination bond between the bidentate ligand and the metal ion, which has a strong impact on the solution stability. It is also well-known that the dissociation of the monodentate (often chlorido) co-ligand (Z) may facilitate the reactions with biological macromolecules such as proteins or DNA. Numerous organoruthenium(II) complexes have been developed and extensively investigated, and some structure-activity relationship analyses have been also conducted to identify the key structural features [2,9,10,11,12,13,14]. These types of comparative studies are highly important for the development of novel, more efficient and selective anticancer complexes.

**Scheme 1:**
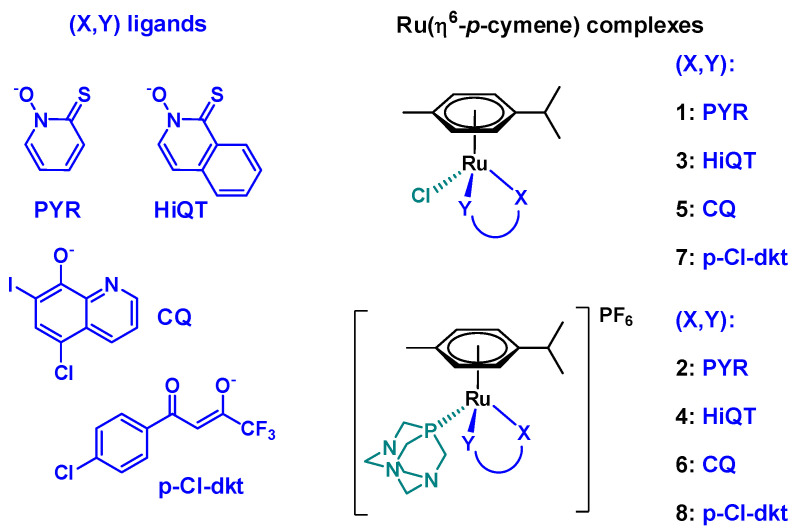
Structures of the studied [Ru(η^6^-*p*-cymene)(X,Y)(Z)] complexes **1**–**8**.

**Figure 1:**
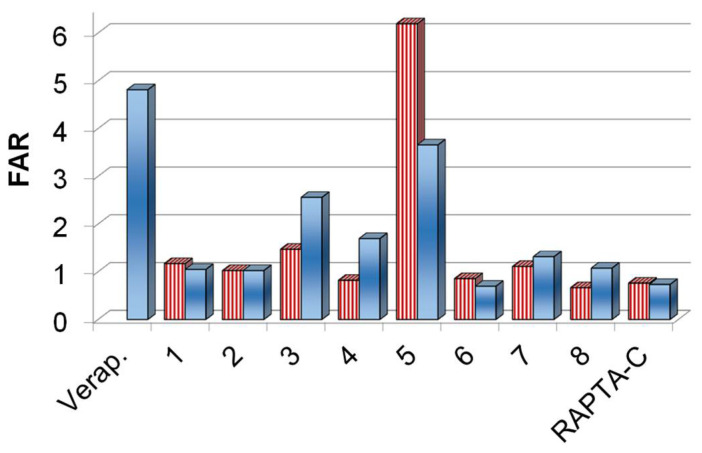
ABCB1 modulating activity on multidrug-resistant Colo 320 colonic adenocarcinoma cells in the presence of complexes **1**–**8**, RAPTA-C at 2 μM (stripped bars) and 20 μM (full bars) concentrations. Verapamil (Verap.) was used as a positive control at 20 μM.

In this work, series of Ru(η^6^-*p*-cymene) complexes with various bidentate ligands were investigated (Figure 1). These compounds were selected from a library of compounds synthesized by the Turel group in last years due to their excellent anticancer potential determined by in vitro cytotoxicity assays on human cancer cell lines. We have thus chosen a *β*-diketone ligand (1-(4-chlorophenyl)-4,4,4-trifluorobutane-1,3-dione (**Hp-Cl-dkt**)) with *O*,*O*-donor set, an 8-hydroxyquinoline (5-chloro-7-iodoquinolin-8-ol, clioquinol (**HCQ**)) bearing *N*,*O*-donor, and two pyrithione-type ligands (1-hydroxypyridine-2(1*H*)-thione (pyrithione, H**PYR**), 2-hydroxyisoquinoline-1(2*H*)-thione (H**HiQT**)) with *O*,*S*-donor set. Either chloride ion or PTA were applied as co-ligands. Synthesis and cytotoxic activity of complexes **1**–**5** and **7**–**8** were reported in our previous works [15,16,17,18,19,20,21]. Additionally, complex **6** was newly prepared to perform a comparative study on cytotoxic, antibacterial (including antichlamydia) and antiviral activity of complexes in relation to their solution stability and reactivity. The differences and similarities in the chemical structure of the selected complexes (Scheme 1) raise questions about the effect of the coordinated bidentate ligands and the substitution of chloride with PTA as co-ligand on the various chemical and biological properties of ruthenium complexes.

## 2. Results and Discussion

### 2.1. Synthesis and Characterization of the Complexes

Complexes **1**–**5** and **7**–**8** were prepared according to published procedures [11,15,16,17,18,19,20,21]. Namely, the neutral chlorido complexes **1**, **3**, **5** and **7** were prepared by the reaction of the corresponding ligand with half-equivalent of dimeric ruthenium precursor [Ru(η^6^-*p*-cymene)(μ-Cl)_2_]_2_ in the presence of sodium methoxide. The PTA-containing complexes **2**, **4**, **6** and **8** were synthesized from the corresponding chlorido compounds using AgPF_6_/NH_4_PF_6_ to remove the chloride ion and upon the addition of PTA phosphine ligand positively charged complexes were obtained as PF_6_^−^ salts. 

Solid state structures **1**–**3**, **5** and **7**–**8** were characterized by single crystal X-ray crystallography [11,15,16,17,18,20,21,22,23]. The aromatic *p*-cymene is π-bounded to the metal ion, and the remaining three coordination sites are occupied by the deprotonated bidentate ligand and the chlorido or PTA co-ligand. The *β*-diketone *O*,*O*-donor **p-Cl-dkt** forms a six-membered ring with ruthenium in which the bonds are delocalized, while a five-membered chelate ring is formed with the other ligands (**PYR**, **HiQT**, **CQ**).

### 2.2. Pharmacological Activity of the Complexes **1**–**8**

Organoruthenium complexes are widely tested for their antitumor activity. As mentioned above some of our investigated complexes in this study have already been tested for their antiproliferation properties towards some cancer cell lines, exhibiting promising activity. However, herein the prepared complexes were tested for the first time against the multidrug resistant Colo 205 and Colo 320 cancer cell lines. It should be noted that multidrug resistance (MDR) is a serious problem in health care regarding microbial infections and cancer. Bacteria and tumor cells are able to develop adaptive strategies for even the most powerful treatments, for this reason the design and screening of possible compounds to reverse or overcome resistance is crucial. The overexpression of MDR membrane transporters is an important resistance mechanism since these transporters extrude harmful agents out of the cells. The inhibition of these efflux pumps is a promising approach to overcome MDR. This is the reason why the compounds were tested against resistant cancer cells (Colo 320) and resistant bacteria (*Staphylococcus aureus*) as well for the inhibition of ABCB1 efflux pump.

Moreover, these complexes have previously also not been tested neither for their antibacterial, antichlamydial nor antiviral properties and as such results present novel knowledge on biological potential of tested compounds. There are several reports on anticancer-antimicrobial dual therapeutic effect, which can be for instance found in the review of Alibek et al. [24]. It is well-known that various infections may take role in initiation and progression of diseases [25]. On the other hand, it is also known that chemotherapeutics commonly weaken the immune system. Therefore, it would be of great benefit if one compound would show various, still synergistic therapeutics effects [26]. In order to investigate whether any of our prepared complexes possess such properties, the selected compounds were tested not only in in vitro cytotoxicity assay, but also for their antibacterial and antiviral activity. 

#### 2.2.1. In Vitro Cytotoxicity of the Complexes **1**–**8** on Cancer cells and Inhibition of the ABCB1 Efflux Pump

Ruthenium complexes are known to exert their anticancer activity acting on multiple molecular targets. Complexes **1–5**, **7–8** were previously screened on selected panels of cancer cell lines and their mode of action was studied by evaluating their interactions with potential enzyme targets related to specific types of cancer as well as molecular mechanisms relevant to metallodrug action such as reactive oxygen species (ROS) generation. It was found, that complexes **1** and **2** trigger early apoptosis by producing ROS resulting in good selectivity towards cancer cells with IC_50_ values in the low micromolar range. The compounds along with a series of complexes containing methylated pyrithione ligands were most effective against lung cancer cells with near nanomolar IC_50_ values. Moreover, compound **1** effectively inhibits aldo-keto reductases AKR1C1 and AKR1C3 resulting in the very low half maximal effective concentration against hormone dependent MCF-7 breast cancer cell line (3.8 μM) [15,16,27]. In addition, another study was conducted where chlorido complex **3** and PTA complex **4** with **HiQT** ligand were screened on an array of cancer cell lines including cisplatin- and adriamycin-resistant strains. Increased lipophilicity of complex **3** resulting from the extension of the aromatic scaffold of the **HiQT** ligand is reflected in even lower IC_50_ values on a similar panel of cancer cell lines, while complex **4** was surprisingly inactive at concentrations up to 50 μM [21]. Complex **5** was also reported to be cytotoxic against a series of human cancer cells and induced caspase-dependent cell death in leukemia cells [17]. Complexes **7** and **8** showed cytotoxicity against ovarian carcinoma CH1 (IC_50_ values 17 and 8 μM, respectively) and the osteosarcoma MG63 cells (IC_50_ values 64 and 41 μM, respectively) [19]. Interestingly, although the chlorido compound **7** was less active in the antiproliferative assay than the PTA complex **8**, complex **7** accumulated more efficiently in the investigated cell lines. Therefore, the differences in the activity can be explained due to various mode of actions, where chlorido complex **7** exerted oxidative stress paralleled by DNA damage induction and apoptotic cell death, whereas for **8** bearing PTA it turned out to inhibit cell cycle in G_0_/G_1_ phase.

Herein, the cytotoxic activity of complexes **1**–**8** was assayed in the chemo-sensitive Colo 205, and the multidrug resistant Colo 320 human colonic adenocarcinoma cell lines, using colorimetric 3-(4,5-dimethylthiazol-2-yl)-2,5-diphenyl-tetrazolium bromide (MTT) test. RAPTA-C and cisplatin were also included for a comparison. Additionally, the cytotoxicity was measured in normal human embryonal lung fibroblast cells (MRC-5). Determined IC_50_ values using 72 h incubation time are collected in Table 1. Complexes **2**, **4** and **6** with the PTA as a co-ligand were non-toxic against the tested cell lines. The same result was obtained for RAPTA-C, which was expected taking into consideration already published data [4]. However, in case of complex **2**, it was previously reported that substitution of chlorido with PTA as a co-ligand did not decrease the activity in case of other types of cancer cells [16]. 

In contrast, for PTA complex **4** with pyrithione ligand with an extended aromaticity it was recently reported to lose the activity once chlorido co-ligand in complex **3** is replaced by PTA [21]. Herein, complex **1** was the most active among the tested compounds; although the benzo-fused analogue complex **3** also shows similar cytotoxicity with a somewhat better selectivity index (i.e., higher ratio of IC_50_ (MRC-5)/IC_50_ (Colo 205 or 320)), and both being more selective than cisplatin. Among complexes **5**–**8**, the PTA-containing complexes **6** and **8** were less cytotoxic than their chlorido counterparts **5** and **7**, as they showed higher IC_50_ values.

Among the tested compounds, chlorido complexes **1**, **3**, **5** and **7** as well as PTA complex **8** exerted significant activity against the multidrug resistant Colo 320 cells. It is important to note, that the resistance of this cell line is primarily mediated by the overexpression of the ABC-transporter P-glycoprotein, which pumps out xenobiotics from the cytosol. Therefore, the effect of the complexes on this efflux pump was monitored via the rhodamine 123 fluorometric assay. Rhodamine 123 is a non-toxic, lipophilic, positively charged mitochondrial specific fluorescent dye and was reported to be a substrate of P-glycoprotein encoded by the human *ABCB1* gene. This dye is membrane-permeable and is rapidly taken up by the cells; therefore, it can be effectively used for the screening of efflux pump inhibiting compounds. The intracellular accumulation of rhodamine 123 was followed by fluorometry at 2 μM and 20 μM concentrations of the compounds in Colo 320 cells (Table S1). Then the fluorescence accumulation ratios (FAR) were calculated (Figure 1) according to the equation given in the Materials and Methods section. Verapamil was used as a reference inhibitor compound. Addition of verapamil to Colo 320 cells increased the fluorescence indicating higher level of accumulation of the fluorescent dye owing to the inhibition of the pump, which resulted in a relatively high FAR value. Most of the tested complexes were characterized by a FAR value around 1. However, higher values were obtained for chlorido complexes **3** and **5** with **HiQT** and **CQ** ligands, respectively suggesting their ABCB1-modulating ability (in these cases FAR values are above 2).

#### 2.2.2. Antibacterial Effect of the Complexes **1**–**8**

The antibacterial activity of the complexes including the well-known RAPTA-C and cisplatin was studied on the Gram-positive *Staphylococcus aureus* and *Enterococcus faecalis* and the Gram-negative *Escherichia coli* and *Klebsiella pneumoniae* strains. The minimum inhibitory concentration (MIC) values are presented in Table 2. All tested Ru complexes had no activity on the Gram-negative bacteria (MIC > 100 μM), and PTA-containing complexes **2**, **4**, **6**, **8**, RAPTA-C as well as cisplatin were not active or had quite low activity against the Gram-positive bacteria. In contrast, chlorido complexes (**1**, **3**, **5**, **7**) displayed clear antibacterial effect on the tested Gram-positive bacteria. This finding agrees with our previous results on organoruthenium chlorido complexes with bromo substituted 8-hydroxyquinolines which also showed good antibacterial potential [23]. Herein, among all tested compounds complex **3** was found to be the most active on Gram-positive organisms. This complex was considerably active on the methicillin-resistant *S. aureus* (MRSA) strain (MIC: 12.5 µM), which is a human pathogen being responsible for several difficult-to-treat hospital-acquired infections [28]. The Gram-positive and Gram-negative bacteria possess different cell wall composition, together with various expression of efflux pumps and membrane proteins, which may contribute to their altered susceptibility to investigated Ru complexes possessing different size, charge and solution chemical properties. In Gram-negative bacteria, the uptake of antibacterial agents depends on the outer membrane (OM). This membrane is an asymmetric bilayer of lipopolysaccharides (LPS) and phospholipids with nonspecific porins and specific channels. In addition, Gram-negative bacteria express efflux transporter proteins consisting of an inner-membrane (IM) pump subunit, a periplasmic adaptor protein and an outer-membrane channel. These transporters can expel structurally unrelated drugs from the bacteria cell. The presence of the outer and inner membranes together with tripartite efflux pumps may result in higher resistance towards antibacterial compounds compared to Gram-positive bacteria.

#### 2.2.3. Antichlamydia Activity of the Complexes **1**–**8**

Among the sexually transmitted diseases *Chlamydia trachomatis*-related infections are the most common. This Gram-negative bacterium can replicate only within a host cell. The various serovars can cause pelvic inflammatory diseases and infertility or lymphogranuloma venereum [29,30]. The antichlamydia activity of the complexes **1**–**8** and RAPTA-C was assayed using human cervix carcinoma HeLa 229 cells. We would like to note that to the best of our knowledge this is the first study describing the effect of organoruthenium(II) compounds against *Chlamydia trachomatis*. As a first step, the cytotoxicity of the complexes was measured by MTT assay in this cell line, and based on the results (Figure S1), the maximum non-toxic concentration of complexes was considered as 100 µM for **1**–**4**, **6** and RAPTA-C, 50 µM for **7** and **8**, and 25 µM for **5**. 

**Figure 2:**
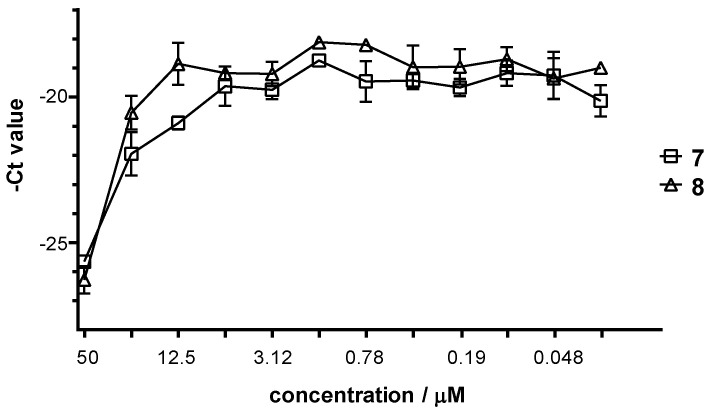
Antibacterial effect of complexes **7** and **8** against *Chlamydia trachomatis*.

Then the Hela cells were infected with *C. trachomatis* at multiplicity of infection (MOI) 0.2, and were treated with different concentrations of the complexes for 24 h. After 48 h post infection, the cells were lysed and the chlamydial growth reducing effect of the complexes was evaluated by comparing the chlamydial genom concentration to that seen on untreated/infected HeLa cells. The *C. trachomatis* DNA concentration was measured by direct quantitative PCR (qPCR). The average cycle threshold (Ct) numbers were calculated and –Ct values are shown at the various complex concentrations in Figure 2 for **7** and **8** (and Figure S2 for the rest of the complexes). The maximum *C. trachomatis* growth corresponded to a DNA concentration of Ct ~19 value as detected by direct qPCR, as well. All of the complexes could inhibit the chlamydial growth on HeLa cells, but the MIC value is higher than 100 µM or higher than the maximum non-toxic concentration. The inhibition curves showed that the most effective complexes were **7** and **8** (Figure 2 and Figure S2), as the growth difference between the treated and untreated cells was ca. 101- and ca. 155-fold, respectively.

Since the growth-related chlamydial DNA concentrations were measured by a qPCR method, the potential direct impact of the complexes on the DNA polymerase of the qPCR [31] was also tested (Figure S3). The obtained results suggest that there is no stimulation or inhibition during the qPCR.

#### 2.2.4. Antiviral Activity of the Complexes **1**–**8**

Human herpes simplex virus-1 (HHSV-1) and preferentially herpes simplex virus-2 (HSV-2) genital infections are common viral sexually transmitted infections. Besides the vesicular lesions of the urogenital and anal regions, human herpes simplex infections may lead to severe complications including encephalitis, meningitis and neonatal herpes infections [32]. The antiviral effect of complexes against HSV-2 was screened using Vero cells (originally isolated from kidney epithelial cells) to host the growing viruses. First, the cytotoxicity of the complexes against Vero cells was assayed (Figure S4), and it was found that complexes **1**–**4** and **7**–**8** did not produce significant toxicity at any of the concentrations used. Maximum cytotoxicity was observed at concentrations of 12.5–100 µM for complexes **5** and **6**, and 100 µM for RAPTA-C, whereas 6.25 µM was considered as maximum non-toxic concentration for **5** and **6**, and 50 µM for RAPTA-C. The maximum non-toxic concentration of complexes **1**–**4** and **7**–**8** were 100 µM.

**Figure 3:**
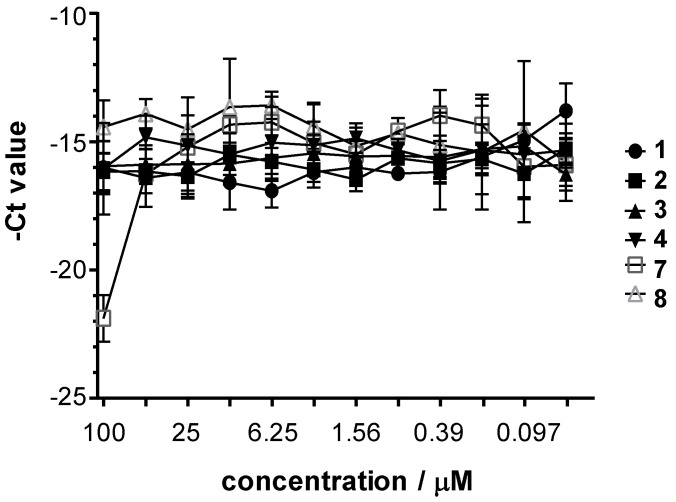
Antiviral effect of complexes **1**–**4** and **7**–**8** against herpes simplex virus-2.

Then Vero cells were infected with HSV-2 (at MOI 0.2) and were treated with the complexes for 24 h. After 24 h post infection, the cells were lysed and the virus yield reducing effect of the compounds was evaluated by comparing the yield to that seen on untreated Vero cells. The HSV-2 DNA concentration was measured by direct qPCR. It should be noted that the possible direct impact of the complexes on the DNA polymerase of the qPCR was also monitored in Vero cells, and no stimulation or inhibition was found. The average –Ct values obtained at the various complex concentrations are shown in Figure 3 for **1**–**4** and **7**–**8** (and Figure S5 for the rest of the complexes). The maximum HSV-2 growth corresponded to a DNA concentration of Ct ~14 value as detected by direct qPCR. Inhibition curves showed that **7** is the most potent complex (~7.89 (dCT = 21.89–14) qPCR cycles difference, which means the growth difference was ca. 237-fold), whilst the other complexes did not inhibit the growth of the HSV-2. The concentration of **7** that decreased the growth of HSV-2 in the cells and the corresponding DNA content by 50% (IC_50_), increased the qPCR Ct value by approximately one cycle. Also, the complex **7** concentration that inhibited the HSV-2 growth by 90% (IC_90_), raised the Ct value by ~3.32 cycles. In the case of complex **7** the IC_50_ was ~25 µM and IC_90_ was between 50–100 µM. 

### 2.3. Solution Speciation of Complexes **1**–**8**

#### 2.3.1. Solution Chemical Properties of *β*-Diketone and 8-Hydroxyquinoline Complexes **5**–**8**

The solution stability of the studied Ru(η^6^-*p*-cymene) complexes was conducted to reveal differences in their solution chemical properties, which may provide explanation for their significantly different biological activity. Solution speciation of numerous Ru(η^6^-*p*-cymene) complexes has already been characterized previously among others including complexes of hydroxamates [33], acetylacetonates [34,35], hydroxy- (thio)pyr(id)ones [36] with *O*,*O*-donor ligands, and 8-hydroxyquinoline derivatives [37,38] or pyridinecarboxylic acids [39] with *N*,*O*-donor set. General equilibrium processes which can take place in solution of the half-sandwich [Ru(η^6^-*p*-cymene)(X,Y)(Z)] complexes are shown in Scheme S1. The herein tested complexes **5** and **6** contain coordinated 8-hydroxyquinolinato ligand **CQ**, while complexes **7** and **8** consist of the *β*-diketonato ligand **p-Cl-dkt** (Scheme 1), for which the binding strength of these ligands is supposed to be significantly different based on the reported stability constants for analogous complexes. 8-Hydroxyquinolines were found to form highly stable complexes with this organometallic cation [37,38], while Ru(η^6^-*p*-cymene) complexes of acetylacetonates were characterized by fairly low stability resulting in the dissociation of the *O,O*-ligand at pH 7.4 in diluted solution [34]. Therefore, the bidentate ligand **CQ** is suggested to be bound rather strongly in the complexes **5**–**6**, while a higher extent of complex decomposition of complexes **7**–**8** bearing the *O,O*-ligand **p-Cl-dkt** is probable.

**Figure 4:**
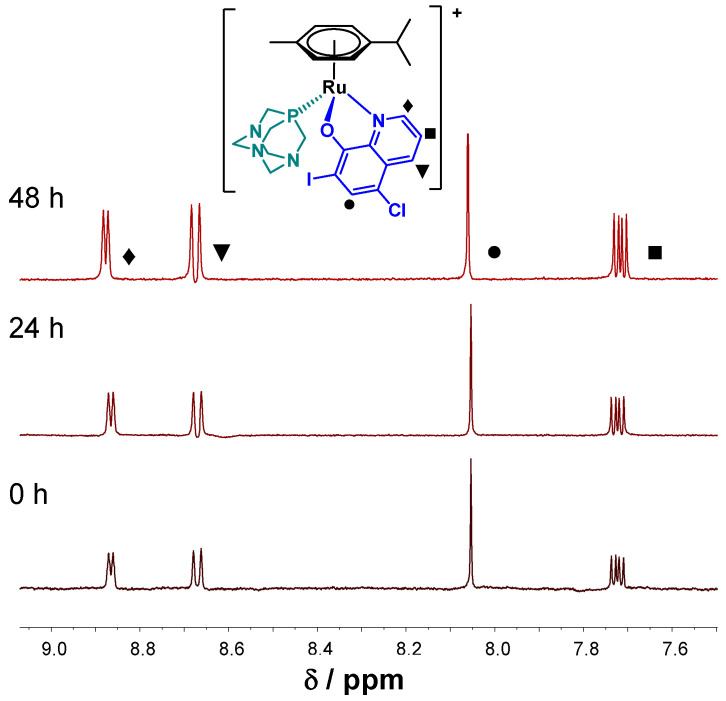
^1^H-NMR spectra of **6** at pH 7.4 (PBS’) in the low field region recorded for the fresh sample, after 24 h and 48 h waiting time. {*T* = 25.0 °C, *c*_complex_ **_6_** = 0.5 mM; 10% (*v*/*v*) DMSO-*d*_6_}.

In order to confirm our prediction, time-dependent UV-visible (UV-vis) spectra were recorded for complexes **5**–**8** at pH 7.4 in aqueous solution (in modified phosphate buffered saline (PBS) buffer—PBS’) and in Eagle’s minimum essential medium (EMEM) used for the cytotoxicity studies in the presence of 2% (*v*/*v*) dimethyl sulfoxide (DMSO). No characteristic UV-vis spectral changes within 24 h were observed for chlorido **5** and PTA complexes **6** with **CQ** ligand in PBS’. However, some precipitation occurred in case of **5** at the applied 50 μM concentration. Additionally, ^1^H-NMR spectra were recorded for more water-soluble complexes **6**–**8**. The ^1^H-NMR spectra of **6** showed no changes during 48 h (Figure 4). On the contrary, UV-vis measurements for both 8-hydroxyquinoline complexes **5**–**6** displayed slow changes in EMEM at the applied 150 μM concentrations (Figure S6). Complex **5** is involved in a relatively fast initial reaction taking place in a 30 min period, where most probably the chlorido co-ligand is exchanged to a medium component (e.g., histidine). Then a similar slow process was seen for both complexes with the absorbance decrease at 425 nm and the appearance of a novel band at 560 nm, which might be the result of the partial loss of the arene ring followed by the oxidation of Ru(II) to Ru(III), as it was also reported for analogous 8-hydroxyquinoline Ru(η^6^-*p*-cymene) complexes [37]. Notably, no indication for arene loss was observed at higher concentration for complex **6** (500 μM) in EMEM according to the ^1^H NMR spectroscopic measurements (not shown). UV-vis spectral changes indicate decomposition of both **p-Cl-dkt** containing complexes **7**–**8** in PBS’ (160 μM), although the PTA co-ligand in complex **8** significantly slowed down this process (Figure S7). The ^1^H-NMR spectra recorded for complex **8** (c = 500 μM, Figure S8) also confirm only a slow transformation in this medium, namely the formation of the chlorido complex **7** and its partial, slow decomposition was found over time. For the complexes these findings confirm the superior solution stability of the 8-hydroxyquinolino chlorido complex **5** over the acetylacetonato chlorido complex **7**, which may explain the lower cytotoxicity and weaker ABCB1 modulating activity of complex **7** compared to complex **5**. On the one hand, the coordination of PTA stabilizes the complexes, which may result in slower co-ligand displacement reaction in comparison to the chlorido complexes. Such kinetics is not beneficial in terms of the biological effect, as it may hinder the coordination of the donor atom of a target macromolecule. All of this is in good agreement with our previous finding for complexes **7** and **8**, where it was reported that PTA delays replacement reactions with soft donor atoms from cellular targets like histidines, cysteines, or methionines and purine bases of DNA [19].

#### 2.3.2. Solution Chemical Properties of Pyrithione-Type Complexes **1**–**4**

Stability constants for complexes **1**–**4** formed with pyrithione (H**PYR**) and its benzo-fused analogue H**HiQT** (Scheme 1) have not been reported yet. Therefore, we aimed to perform an in-depth study on the solution speciation of complexes **1**–**4** (Scheme 1), since among them **1** and **3** proved to have remarkable anticancer activity and antibacterial effect on Gram-positive bacteria (Table 1 and Table 2). The solution speciation studies of complexes **1**–**4** were performed using UV-vis spectrophotometry and pH-potentiometry in the presence of 200 mM chloride ions. However, measurements were limited by the insufficient water solubility of ligand H**HiQT** and the oxygen-sensitivity of both pyrithiones. The pK_a_ values determined for the ligands are shown in Table 3, where values for H**PYR** are in a good agreement with previously reported data [40]. In case of H**HiQT**, the pK_a_ could be determined only by UV-vis titrations under argon atmosphere, although, oxidation was observed at pH > 5.5 due to O_2_ traces in the sample.

**Figure 5:**
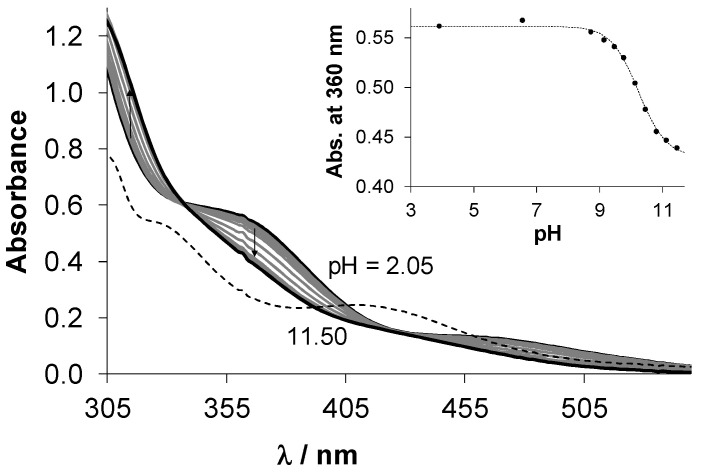
UV-vis absorption spectra recorded for complex **1** in the presence of 200 mM chloride ions at various pH values (solid lines), in addition to the summed spectra of the unbound ligand pyrithione and [Ru(η^6^-*p*-cymene)(H_2_O)_3_]^2+^ at pH 2 (dashed line) using the same concentration of the complex, ligand and metal precursor in the samples. Inserted figure shows the absorbance values of complex **1** at 360 nm plotted against the pH. {*T* = 25.0 °C, *I* = 0.2 M KCl, *c*_complex_ **_1_** = 250 μM; *l* = 1 cm}.

In order to obtain information about the stability of the isolated complexes **1**–**4**, pH-dependence of their UV-vis spectra was monitored in the pH range 2–11.5. In case of **1** and **3**, UV-vis spectra recorded at pH 2 revealed the formation of highly stable complexes as the spectra recorded for the metal precursor–ligand mixture (1:1) were significantly different (for **1** see Figure 5). Then, the ^1^H-NMR spectrum was recorded after 24 h where the pH of the solution of complex **1** was decreased to 1.0 to enforce complex dissociation. Figure S9 shows only a minor fraction of unbound Ru(η^6^-*p*-cymene) (<3%), which confirms the high solution stability of complex **1**. UV-vis spectra of complexes **2** and **4** stayed unchanged in pH range 2–11 during the monitored 24 h period (not shown). On the contrary, spectra recorded for complexes **1** and **3** displayed significant changes in the basic pH range (for **1** see Figure 5). This process is fast and the appearance of isosbestic points also suggest that these spectral changes are due to the coordination of hydroxide ions replacing the co-ligand (see Scheme S1 for the deprotonation of the aqua ligand). For this process, pK_a_ values were calculated for both complexes from pH-potentiometric and UV-vis titration data (Table 3), which reflect that hydroxido species are not formed at physiological pH (notably, these pK_a_ values are valid only in the presence of 200 mM chloride ions, thus, are considered as conditional constants). Complexes **1** and **3** were found to be stable at pH 7.4 in various media (e.g., phosphate buffered saline (PBS), RPMI-1640 cell culture medium) and only minor release of *p*-cymene was observed after 24 h in case of **1** and **3**, respectively [21,41].

The high solution stability of **1** and **3** hindered the direct and accurate calculation of their formation constants, and a logK > 8 value is suggested on the basis of the pH-potentiometric data. In order to compare the stability of **1** and **3** at physiological pH, a serial dilution was made and UV-vis spectra were recorded in a wide concentration range (10 μM–3 mM) (Figure S10). The calculated molar absorbance (ε) values change more significantly with decreasing the concentration in case of complex **1** suggesting the somewhat higher stability of its analogue complex **3** containing pyrithione with an extended aromaticity. We attempted to determine the stability constants for **1** and **3** using 1,10-phenantroline as a competitor ligand, although it failed due to the arene loss of the complexes (see details in Figure S11). 

The lipophilicity of chlorido complexes **1** and **3** was characterized via the conventional shake-flask method (PTA complexes **2** and **4** were found to be too lipophilic and could not be measured), and distribution coefficients (D_7.4_) are collected at pH 7.4 at different chloride ion concentrations in Table 4. Chloride ion concentrations of 4, 24 and 100 mM were chosen according to the chloride content of the nucleus, cytosol and blood serum, respectively. The lipophilicity of both tested complexes is enhanced with increasing chloride ion concentrations, most probably due to higher fraction of the neutral chlorinated complexes over the positively charged aqua complexes. Complex **3** is more lipophilic than **1** due to the presence of the additionally condensed benzene ring. Then parallel artificial membrane permeability assay (PAMPA) was used to estimate the ability of the complexes to penetrate membranes by passive diffusion and the effective passive permeability coefficients (*P*_eff_) obtained for complexes **1**–**3** are shown in Table 4 (in case of **4**, there are no data due to formation of precipitate). The assay was performed at pH 7.4 in the presence of 100 mM chloride ion and it shows the permeability order: **3** > **2** > **1**. Therefore, both the conjugation of the benzene ring and the coordination of PTA increase the membrane permeability of the complexes. Despite higher lipophilicity and membrane permeability of PTA complexes in case of the acetylacetonato type **p-Cl-dkt** complexes (**7**, **8**), higher accumulation of the chlorido complex **7** was reported [19]. 

Since it was found that the chloride/water exchange has an impact on the lipophilicity of the complexes, we attempted to characterize this equilibrium process (Scheme S1) as it was done for other half-sandwich organometallic complexes [37,38,42]. Unfortunately, the chloride/water exchange in these complexes was not accompanied by measurable changes in the UV-vis spectra, unlike the PTA/Cl^−^ exchange (Scheme S1). Thus, the possibility of the replacement of PTA in complexes **2** and **4** was monitored spectrophotometrically upon the addition of chloride ions. However, even the addition of a huge excess of chloride ion (>10^4^) did not result in spectral changes, thus this halide ion cannot compete efficiently with PTA. The reverse reaction was also followed (Figures S12 and S13), and practically the quantitative replacement of the chlorido ligand in complexes **1** and **3** was observed indicating the much stronger binding of PTA compared to Cl^−^.

### 2.4. Interaction of Complexes **1**–**4** with Human Serum Albumin

**Figure 6:**
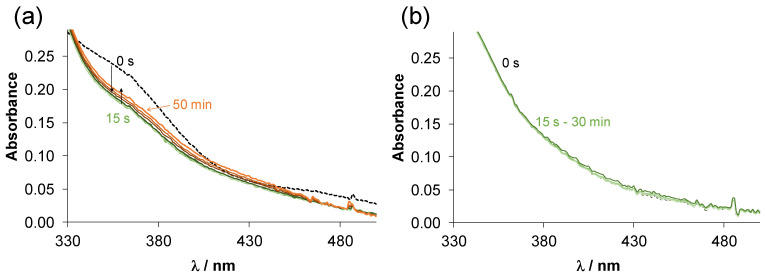
Time-dependence of UV-vis absorption spectra recorded for (**a**) complex **1** and (**b**) complex **2** in the presence of 0.5 equiv. HSA at pH = 7.4. {*T* = 25.0 °C, *c*_complex_ = 100 μM, pH = 7.40 PBS’ buffer; *l* = 1 cm}.

**Figure 7:**
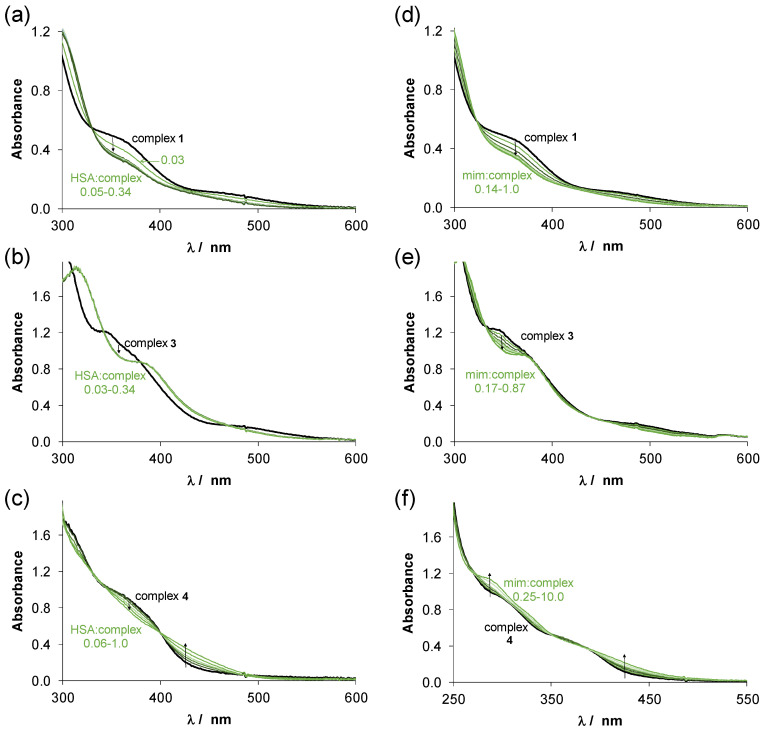
UV-vis absorption spectra recorded for (**a**,**d**) complex **1**, (**b**,**e**) complex **3** and (**c**,**f**) complex **4** in the presence of various equivalents of HSA or mim, respectively at pH = 7.4. The numbers indicate the range of the *c*_HSA_/*c*_complex_ or *c*_mim_/*c*_complex_ ratios. In case of PTA complexes 24 h incubation time was used. {*T* = 25.0 °C, pH = 7.40 PBS’ buffer, *c*_complex_ = 210 μM, *c*_HSA_= 6.3–71.4 μM or *c*_mim_ = 29–210 μM (**a**,**b**,**d**,**e**); *c*_complex_ = 116 μM, *c*_HSA_= 6.3–71.4 μM or *c*_mim_ = 29–1160 μM with 8% (*v*/*v*) DMSO (**c**,**f**); *l* = 1 cm}.

Human serum albumin (HSA) is the most abundant protein in the blood and exerts an important role in the transport and distribution of exogenous and endogenous molecules. Additionally, binding of antitumor compounds to HSA is of considerable interest due to the enhanced permeability and retention effect [43]. Complexes **1**–**4** were selected for a more detailed study. The binding of these complexes on HSA was monitored by UV-vis spectrophotometry and fluorometry in a PBS’ containing 100 mM chloride ions according to the blood serum. First, the binding was followed in time spectrophotometrically, and representative UV-vis spectra were recorded at half equivalent of HSA as shown in Figure 6 for complexes **1** and **2** (complexes **3** and **4** behaved similarly to **1** and **2**, respectively). The spectra recorded for **1** (Figure 6a) displayed a bi-phasic binding profile, namely, a very fast process was followed by a much slower one. For the Ru(η^6^-*p*-cymene) triaqua cation, it was suggested that the binding is completed in the first step, and the subsequent slow and minor changes are due to some structural rearrangement of the coordination sphere around the metal center [44]. While, the spectra in case of **2** were almost unchanged in the presence of the protein (Figure 6b) suggesting that there is only a minimal measurable interaction between them during the monitored time (30 min) at the applied concentrations (100 μM complex, 50 μM HSA). Then, spectra were recorded for the complexes at various complex-to-HSA ratios (Figure 7a–c). In the case of the chlorido complexes titrations were done with HSA, due to the fast binding; otherwise, 24 h incubation time was applied (in case of PTA complexes). The spectra became constant after the addition of rather low equivalents of the protein (<1) indicating that more than one complex is bound on one HSA molecule. However, in case of **4** a much higher amount of HSA was needed to reach the constant spectra. Based on the spectral changes, the binding affinity of the complexes gives the following order: **3** > **1** > **4** > **2**. 

Thus, the chlorido complexes are bound stronger (and faster) than the PTA containing compounds, as in the latter case the strongly bounded PTA should be replaced by the coordinating donor atom of the protein. Binding data of complexes **1** and **2** herein correlate well with data already published for these complexes to HSA and bovine serum albumine (BSA) by HPLC-ICP-MS [41] and AAS [16] methods, respecitvely, where chlorido complex **1** was bound to HSA/BSA in greater extent than PTA complex **2**.

Organometallic half-sandwich complexes are often bound to HSA via coordinative bounds [44,45] and His imidazoles are suggested as main binding sites among the potential donors of other residues such as Glu, Cys, Asp. In our previous work, the fast interaction of complexes **7** and **8** with thiol-containing low molecular mass endogenous compounds Cys, *N*-acetylcysteine and glutathione was found on the basis of a mass spectrometry study [46]. The donor atoms generally coordinate at the site of the monodentate co-ligand, however, they can also displace the weakly bound bidentate ligand as well. Herein, interaction of the complexes **1**–**4** towards 1-methylimidazole (mim), a monodentate binding model of HSA was measured by UV-vis (Figure 7d–f). 

The reactions in all cases were very fast (except complex **2** and **4**), and the observed spectral changes were similar to those obtained with HSA (Figure 7a–c). There was no indication for the replacement of the bidentate ligand by 1-methylimidazole. Considering that the latter ligand binds at the coordination site of the co-ligand, maximum one complex is able to be bound to 1-methylimidazole. However, the constant spectra upon addition of 1-methylimidazole to the complex **4** could only be reached at high excess of 1-methylimidazole due to its lower tendency to interact with this model (and with HSA as well). Based on these spectral changes, conditional binding constants were calculated for the formation of the 1-methylimidazole adducts. Log*K*’ = 4.16 ± 0.45, 4.18 ± 0.27 and 3.64 ± 0.02 were obtained for complexes **1**, **3** and **4**, respectively (notably, complexes **1** and **3** were bound to 1-methylimidazole almost quantitatively). These results also confirm stronger binding of the chlorido complexes over the PTA species. The protecting role of PTA in ligand-exchange processes for the complex pair **7** and **8** was also reported regarding their interaction with thiols or protein NCp7 [46,47]. 

**Figure 8:**
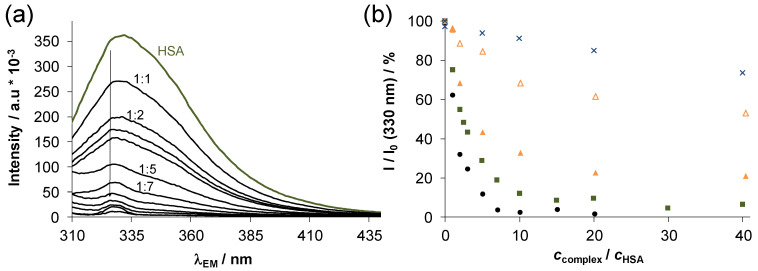
(**a**) Fluorescence emission spectra obtained for HSA titrated by complex **1** using λ_EX_ = 295 nm. (**b**) Intensity changes (%) at 330 nm at the various HSA-to-complex ratios for complex **1** (■), complex **2** (×), complex **3** (●) complex **4** (∆; ▲ (batch samples using 48 h equilibration time)). {*T* = 25.0 °C, pH = 7.40 PBS’ buffer, *c*_HSA_= 1 μM}.

Steady-state spectrofluorometric measurements were also performed to assay the binding interactions at the binding pockets of HSA located in the IIA and IIIA subdomains. His residues are found nearby at both binding sites [48], and covalent binding is possible here. Trp-214 quenching and site marker displacement experiments were conducted as reported in our former works [44,49]. Warfarin (WF) was applied as site I marker and dansyl glycine (DG) was used for site II. Representative emission spectra for Trp-214 quenching in case of **1** are shown in Figure 8a, and changes in the intensities at the emission maximum are compared for **1**–**4** in Figure 8b. Based on the spectral changes conditional binding constants were computed for both of the quenching and site marker displacement assays (Table 5). 

As the binding of the chlorido complexes was found to be relatively fast, the quenching experiment was performed as titration; however, in case of complex **4** the batch method and 48 h equilibration time was used which provided a higher constant indicating that the displacement of PTA is a slow process. The log*K*_Q_’ quenching constants reveal the significantly stronger binding of complexes **3** and **1** compared to **4** and **2**. Complex **2** is hardly bound at site I, while binding of **3** and **1** is considered as a strong interaction here. Displacement experiments were done only with these strongly bound compounds, and the determined constants (Table 5) show that they interact at both binding sites of HSA.

## 3. Materials and Methods

### 3.1. Chemicals

Chemicals and solvents used for the synthesis were purchased from commercial suppliers: Fluorochem (Hadfield, UK), Sigma Aldrich (St. Louis, MO, USA), Strem Chemicals, Inc. (Newburyport, MA, USA) and used as received, except for the phosphine ligand PTA, which was synthesized as reported [50]. [Ru(η^6^-*p*-cymene)(μ_2_-Cl)Cl]_2_, H**PYR**, H**HiQT**, phen, mim, KCl, HCl, KOH, 4,4-dimethyl-4-silapentane-1-sulfonic acid (DSS), NaH_2_PO_4_, Na_2_HPO_4_, KH_2_PO_4_, DMSO and HSA (A8763, essentially globulin free) were purchased from Sigma-Aldrich in puriss quality. Ultrapure Milli-Q water was used for sample preparation. PBS buffer and its modified version (PBS’) were used to adjust pH 7.4. PBS’ contains 12 mM Na_2_HPO_4_, 3 mM KH_2_PO_4_, 1.5 mM KCl and 100.5 mM NaCl; and the concentration of the K^+^, Na^+^ and Cl^−^ ions corresponds to that of the human blood serum. Stock solutions of HSA, WF and DG were prepared and their concentrations were determined as described previously [49,51].

### 3.2. Synthesis and Characterization of Complexes

Complexes **1**–**5** and **7**–**8** were synthesized and characterized in our former publications [11,15,16,17,18,19,20,21], while complex **6** was newly prepared. Complex **6** was characterized by NMR spectroscopy on an Avance III 500 spectrometer (Bruker, Billerica, MA, USA). ^1^H-NMR spectra were recorded at room temperature at 500 MHz, with chemical shifts (**δ**) expressed in ppm and coupling constants (*J*) in Hz. ^1^H-NMR spectra were referenced to the residual solvents relative to external standard. Multiplicities are abbreviated as s = singlet, d = doublet, dd = doublet of doublets, ddd = doublet of doublets of doublets, dt = doublet of triplets and m = multiplet. High-resolution mass spectrum (HRMS) was recorded on a 6224 Accurate Mass TOF LC/MS instrument (Agilent, Santa Clara, CA, USA). IR spectra was obtained with a Bruker FTIR Alpha Platinum ATR spectrometer. UV-vis spectrum for the characterization was recorded with a LAMBDA 750 UV-vis-near IR spectrophotometer (PerkinElmer, Waltham, MA, USA). Elemental analysis (C, H, N) was carried out on a Perkin-Elmer 2400 II instrument. 

For the synthesis of complex **6**, the chlorido complex **5** (30 mg, 0.052 mmol, 1 mol. equiv.) and AgPF_6_ (16 mg, 0.063 mmol) in a molar ratio 1:1.2 were suspended in methanol in a brown round-bottom flask and refluxed for 20 min. A 1.5 molar equivalent amount of PTA (12 mg, 0.078 mmol), was first dissolved in 10 mL of CHCl_3_ and slowly added to the reaction mixture, which was further refluxed for 2 h. Afterwards, the solvents were removed using a rotary evaporator, the oily residue was re-dissolved in CH_2_Cl_2_ and the reaction mixture was filtered over Celite to remove the precipitated AgCl. The clear solution was concentrated to 5–10 mL, and 10 mL of cold n-hexane was added to precipitate respective product **6**. The yellow product was filtered and dried overnight at 45 °C. Yield: m = 27.2 mg, η = 62%. ^1^H-NMR (DMSO-*d*_6_): δ 8.79 (d, *J* = 5.1 Hz, 1H, C^2^H), 8.53 (dt, *J* = 8.6, 0.9 Hz, 1H, C^4^H), 7.96 (s, 1H, C^6^H), 7.76 (dd, *J* = 8.7, 5.0 Hz, 1H, C^3^H), 6.27 (dd, *J* = 6.0, 2.1 Hz, 1H, Ar-H cym), 6.18 (dd, *J* = 13.6, 6.3 Hz, 2H, Ar-H cym), 5.99 (d, *J* = 6.0 Hz, 1H, Ar-H cym), 4.30 (s, 6H, PTA), 3.78 (ddd, *J* = 46.1, 14.8, 2.7 Hz, 6H, PTA), 2.79–2.70 (m, 1H, Ar-CH(CH_3_)_2_ cym), 2.23 (s, 3H, Ar-CH_3_ cym), 1.29 (d, *J* = 6.9 Hz, 3H, Ar-CH_3_ cym), 1.21 (d, *J* = 6.9 Hz, 3H, Ar-CH_3_ cym). Selected IR peaks (cm^−1^, ATR): 3081, 2980, 2937, 2905, 1671, 1576, 1547, 1485, 1439, 1389, 1367, 1286, 1243, 1049, 1032, 971, 945, 900, 883, 865, 821, 802, 783, 742, 711, 658, 633. ESI-HRMS (CH_3_CN) *m*/*z* [M − PTA − PF_6_]^+^: exp. 539. 9165, calc. 539.9155; [M − PF_6_]^+^ exp. 696.9934, calc. 696.9918. UV-vis (λ [nm] (ε [M^−1^ cm^−1^]) c = 1 × 10^−4^ M, CHCl_3_: 293 (24,600), 371 (5500), 450 (4700). Elemental analysis CHN (%) for C_25_H_30_ClF_6_IN_4_OP_2_Ru: calc. C, 35.67; H, 3.59; N, 6.65; found C, 35.11; H, 3.61; N, 6.97.

### 3.3. Solution Studies: pH-potentiometry, UV-vis Spectrophotometry and ^1^H NMR Spectroscopy

pK_a_ values and the accurate concentration and of the ligand stock solutions were determined by pH-potentiometric titrations with the use of the computer program Hyperquad2013 [52]. pH-potentiometric measurements were carried out at 25.0 ± 0.1 °C in water and at a constant ionic strength of 0.20 M KCl. Titrations were performed in a carbonate-free KOH solution (0.20 M). The exact concentration of HCl and KOH solutions was determined by pH-potentiometric titrations. A 710A pH-meter (Thermo Scientific Orion, Waltham, MA, USA) equipped with a type 6.0234.100 combined electrode (Metrohm, Herisau, Switzerland) and a Metrohm 665 Dosimat burette were used for the pH-potentiometric measurements. The electrode system was calibrated to the pH = −log[H+] scale as suggested by Irving et al. [53]. The average water ionization constant, pK_w_, was determined as 13.76 ± 0.01 at 25.0 °C, which is in accordance to literature [54]. The pH-potentiometric titrations were performed in the pH range between 2.0 and 11.5.

An Agilent Cary 8454 diode array spectrophotometer was used to record the UV-vis spectra in the interval 200–800 nm. The path length (l) was usually 1 cm, and 2, 5, 20 or 50 mm cells were used occasionally. Equilibrium constants (proton dissociation, stability constants, conditional stability constants (*K*’_7.4_) for the formation of mim adducts) and the individual spectra of the species were calculated with the computer program PSEQUAD [55]. The spectrophotometric titrations were performed in aqueous solution of the complexes at 25.0 ± 0.1 °C at an ionic strength of 0.20 M (KCl). Spectrum of the solution of 200 μM complex upon addition of one or two equivalents of its own ligand or phen was also measured at pH 7.4. UV-vis spectra recorded for the complexes as a function of chloride concentrations or PTA (in case of the chlorido complexes) were used to investigate the co-ligand exchange processes at pH 7.4. Interaction with mim was followed spectrophotometrically at pH 7.4 (20 mM phosphate buffer) at 102 mM chloride ion concentrations. In some cases, the measurements were performed in the presence of 1–2% (Cl^−^/PTA exchange reactions) or 6–8% (*v*/*v*) (interaction of mim with complexes **2**, **4**) DMSO. Stability of complexes **5**–**8** was also monitored over time (up to 48 h) at 150–160 μM in PBS’ or in EMEM (with 2–6% (*v*/*v*) DMSO) by UV-vis spectrophotometry. 

^1^H-NMR stability studies were carried out on a Bruker Avance III HD 500 MHz instrument. All ^1^H-NMR spectra were recorded on samples containing 10% (*v*/*v*) D_2_O with the WATERGATE water suppression pulse scheme using DSS internal standard. Stability of complexes **6**–**8** was checked over time by ^1^H-NMR spectroscopy at 500 μM in PBS’ or in EMEM (with 10% (*v*/*v*) DMSO-d_6_).

### 3.4. Lipophilicity and PAMPA Measurements

Distribution coefficients at physiological pH (D_7.4_) of the complexes were determined by the traditional shake-flask method in n-octanol/buffered aqueous solution at pH 7.40 (20 mM phosphate buffer) at various chloride concentrations and at 25.0 ± 0.2 °C using UV-vis detection as described in our former works [37,38,42]. PAMPA was applied for the complexes with a Gentest pre-coated PAMPA Plate System (Corning, Corning, NY, USA) [56]. Briefly, a 96-well filter plate was used as the permeation acceptor and the 96-well bottom plate was used as the permeation donor. For simplicity, PBS was used both as donor and acceptor buffer throughout this study. The initial donor solutions were prepared by diluting stock solutions in PBS (300 μM (complexes **1** and **3**) or 65 μM (complexes **2** and **4**). Donor plate was filled with 300 μL of the donor solutions (containing the test compounds). Each well of the filter plate contained 200 μL buffered solution as acceptor phase. The resulting ‘sandwich’ was protected with parafilm to prevent evaporation and incubated at room temperature for 5 h. Then, solutions from the donor and acceptor wells were transferred to Eppendorf tubes and their UV-vis spectra were recorded to determine the concentration of the components. *P*_eff_ values were calculated according to the equation reported by Yu et al. [57].

### 3.5. HSA Binding Studies: UV-vis and Fluorometry

UV-vis spectra were recorded using an Agilent Cary 8454 diode array spectrophotometer for HSA containing samples in which the complex concentrations were 200 μM or 100 μM and 0–1 equivalents of HSA was added. The measurements were performed at 25.0 ± 0.2 °C at pH 7.4 using PBS’. The samples contained 6–8% (*v*/*v*) DMSO in case of complexes **2** and **4**.

Fluorescence studies were performed using a Fluoromax (Horiba Jobin Yvon, Kyoto, Japan) fluorometer in 1 × 1 cm quartz cells. All samples contained 1 μM HSA and various HSA-to-metal complex ratios (up to complex:HSA = 1:40) were used. Site marker displacement experiments were also carried out, and in the samples the HSA-to-site marker (WF or DG) ratio was 1:1 and the concentration of the complex compounds was varied. For the Trp quenching, the WF and DG displacement experiments λ_EX_ = 295, 310 and 335 nm was applied and emission spectra were recorded spectra in the wavelength range 310–450, 320–500 and 420–600 nm, respectively. Computer program HypSpec [51] was utilized for calculation of binding constants (*K*’) for HSA–metal complex adducts similar to the approach described in our former works [43,48]. Calculations were always based on data obtained from at least two independent measurements. Corrections for self-absorbance and inner filter effect were done as described in our former works [44,49] using the formula suggested by Lakowicz [58].

### 3.6. In Vitro Cytotoxicity Studies

#### 3.6.1. Cell Lines and Culture Conditions

Human colonic adenocarcinoma cell lines Colo 205 doxorubicin-sensitive (ATCC-CCL-222) and Colo 320/MDR-LRP multidrug resistant expressing ABCB1 (MDR1)-LRP (ATCC-CCL-220.1) were purchased from LGC Promochem (Teddington, UK). The cells were cultured in RPMI 1640 medium supplemented with 10% heat-inactivated fetal bovine serum, 2 mM l-glutamine, 1 mM Na-pyruvate and 100 mM 4-(2-hydroxyethyl)-1-piperazineethanesulfonic acid (HEPES, Sigma, Steinheim, Germany). Cell lines were incubated at 37 °C, in a 5% CO_2_, 95% air atmosphere. The semi-adherent human colon cancer cells were detached with Trypsin-Versene (EDTA, Sigma, Steinheim, Germany) solution for 5 min at 37 °C. MRC-5 human embryonal lung fibroblast cell lines (ATCC CCL-171) were purchased from LGC Promochem. The cell line was cultured in EMEM (containing 4.5 g/L glucose) (Sigma, Steinheim, Germany) supplemented with a non-essential amino acid mixture, a selection of vitamins and 10% heat-inactivated fetal bovine serum (FBS, Sigma, Steinheim, Germany). The cell lines were incubated at 37 °C, in a 5% CO_2_, 95% air atmosphere.

HeLa 229 cells (ATCC) and Vero cells (ATCC) were cultured in minimal essential medium (MEM) (Sigma, Steinheim, Germany) with Earle salts supplemented with 10% heat-inactivated fetal bovine serum (FBS) (Gibco; ThermoFisher Scientific, Inc., Waltham, MA, USA), 2 mM L-glutamine, 1× non-essential amino acids, 8 mM HEPES, 25 μg/mL gentamycin and 1 µg/mL fungisone. The cells were incubated for 1 h at room temperature to reduce the edge effect and then overnight at 37 °C, 5% CO_2_ to obtain a 90% confluent cell layer.

#### 3.6.2. MTT Assay for Cytotoxic Effect

In the study MRC-5 non-cancerous human embryonic lung fibroblast and human colonic adenocarcinoma cell lines (doxorubicin-sensitive Colo 205 and multidrug resistant Colo 320 colonic adenocarcinoma cells) were used to determine the effect of compounds on cell growth. The effects of increasing concentrations of compounds on cell growth were tested in 96-well flat-bottomed microtiter plates. The stock solutions of the compounds were prepared in DMSO, and in the final samples the DMSO content was always lower than 1%. The compounds were diluted in a volume of 100 μL of medium.

The adherent human embryonal lung fibroblast cells were cultured in 96-well flat-bottomed microtiter plates, using EMEM supplemented with 10% heat-inactivated fetal bovine serum. The density of the cells was adjusted to 1 × 10^4^ cells in 100 μL per well, the cells were seeded for 24 h at 37 °C, 5% CO_2_, then the medium was removed from the plates containing the cells, and the dilutions of compounds previously made in a separate plate were added to the cells in 200 μL. In case of the colonic adenocarcinoma cells, the two-fold serial dilutions of compounds were prepared in 100 μL of RPMI 1640, horizontally. The semi-adherent colonic adenocarcinoma cells were treated with Trypsin-Versene (EDTA) solution. They were adjusted to a density of 1 × 10^4^ cells in 100 μL of RPMI 1640 medium, and were added to each well, with the exception of the medium control wells. The final volume of the wells containing compounds and cells was 200 μL. 

The culture plates were incubated at 37 °C for 72 h; at the end of the incubation period, 20 μL of MTT (Sigma) solution (from a stock solution of 5 mg/mL) were added to each well. After incubation at 37 °C for 4 h, 100 μL of sodium dodecyl sulfate (SDS) (Sigma) solution (10% in 0.01 M HCl) were added to each well and the plates were further incubated at 37 °C overnight. Cell growth was determined by measuring the optical density (OD) at 540/630 nm with Multiscan EX ELISA reader (Thermo Labsystems, Cheshire, WA, USA). Inhibition of the cell growth (expressed as IC_50_: inhibitory concentration that reduces by 50% the growth of the cells exposed to the tested compounds) was determined from the sigmoid curve where 100 − ((OD_sample_ − OD_medium control_)/(OD_cell control_ − OD_medium control_)) × 100 values were plotted against the logarithm of compound concentrations. Curves were fitted by Prism software (GraphPad Software Inc., San Diego, CA, USA) using the sigmoidal dose-response model (comparing variable and fixed slopes). The IC_50_ values were obtained from at least 3 independent experiments.

The maximum non-toxic concentration of the complexes on HeLa 229 (ATCC) and Vero cells was determined by MTT assay as well, similarly as shown vide supra for the MRC-5, Colo 205 and Colo 320 cells. The density of cells was 4 × 10^4^ cells/well in 100 µL of MEM with Earle salts supplemented with 10% heat-inactivated FBS, 2 mM l-glutamine, 1× MEM vitamins, 1× non-essential amino acids, 0.005% Na-pyruvate, 25 µg/mL gentamycin, 1 µg/mL fungisone). The plates were incubated for 1 h room temperature and then overnight at 37 °C, 5% CO_2_. Next day, when the cells reached a ~90% confluency, the medium was supplemented with the serial 2-fold dilutions of the complexes in three parallel wells for each concentration. Concentration ranges of 0.048–100 μM for each complex were tested. After 24 h incubation for Vero cells and 48 h incubation for HeLa cells, 10 µL of the MTT (Sigma, Steinheim, Germany) labelling reagent (final concentration 0.5 mg/mL) was added to each well and the plate was incubated for 4 h at 37 °C, 5% CO_2_. After the incubation, 100 µL of the solubilisation solution (10% SDS in 1 M HCl) was added into each well, and the plate was allowed to stand overnight in the incubator at 37 °C, 5% CO_2_. Cell growth was determined using the same approach as shown vide supra.

### 3.7. Rhodamine 123 Uptake/Retention Fluorescence Assay

The inhibition of the cancer multidrug efflux pump ABCB1 by the tested compounds was evaluated using flow cytometry, measuring the retention of rhodamine 123 by ABCB1 (P-glycoprotein) in Colo 320 colonic adenocarcinoma cells overexpressing the ABCB1 transporter. This fluorescence-based detection system uses verapamil (a first-generation, competitive EPI) as reference inhibitor. Briefly, cell numbers of colonic adenocarcinoma cells were adjusted to 2 × 10^6^ cells/mL, re-suspended in serum-free RPMI-1640 medium and distributed in 0.5 mL aliquots into Eppendorf centrifuge tubes. The tested compounds were added at different concentrations (2 and 20 µM; from 1 and 10 mM stock solutions, respectively) and the samples were incubated for 10 min at room temperature. Verapamil (EGIS Hungarian Pharmaceutical Company, Budapest, Hungary) was applied as positive control (20 µM final concentration) and DMSO was applied as solvent control (at 2% (*v*/*v*). The fluorescent rhodamine 123 was used to monitor the inhibition of the ABCB1 efflux pump (λ_EX/EM_ = 505/534 nm). Therefore, as the next step 10 µL (5.2 µM final concentration) of rhodamine 123 (Sigma) was added to the samples and the cells were incubated for 20 min at 37 °C. After the incubation period, the cells were washed twice and re-suspended in 0.5 mL PBS for analysis. The fluorescence of the gated cell population was measured with a Partec CyFlow^®^ flow cytometer (Partec, Münster, Germany). The percentage of mean fluorescence intensity was calculated for the treated cells as compared with the untreated cells. The results were obtained from a representative flow cytometry experiment in which at least 2 × 10^4^ individual cells of the overall population were evaluated for the rhodamine 123 retained inside the cells. The fluorescence activity ratio (FAR) was calculated based on the following equation, which relates the fluorescence intensities measured on the MDR and parental cells and their controls:

$$\mathrm{FAR}=\frac{{\mathrm{Colo}\;320}_{\mathrm{treated}}{\;/\;\mathrm{Colo}\;320}_{\mathrm{control}}}{{\mathrm{Colo}\;205}_{\mathrm{treated}}{\;/\;\mathrm{Colo}\;205}_{\mathrm{control}}}$$

The parameters evaluated from flow cytometric experiments are forward scatter count (FSC, provides information about cell size); side scatter count (SSC, proportional to cell granularity or internal complexity); mean fluorescence of the cells (FL-1) and the computed FAR and FAR quotients (Table S1).

### 3.8. Antibacterial Effect: Bacterial Cell Culture and MIC Determination

Wild-type *Escherichia coli* K-12 AG100 strain [argE3 thi-1 rpsL xyl mtl Δ(gal-uvrB) supE44] expressing the AcrAB TolC EP at its basal level and *Klebsiella pneumoniae* ATCC 49,619 Gram-negative strains were studied in the experiments. *Staphylococcus aureus* ATCC 43,300 methicillin-resistant (MRSA) strain and *Enterococcus faecalis* ATCC 29,212 strain were used as Gram-positive strains in the assays. MIC values of the complexes were determined in 96-well plates based on the Clinical and Laboratory Standard Institute guidelines (CLSI guidelines) [59]. The stock solutions of the compounds (dissolved in DMSO using 10 mM concentration) were diluted in 100 μL of Mueller Hinton Broth. Then 10^−4^ dilution of an overnight bacterial culture in 100 μL of medium was added to each well, with the exception of the medium control wells. The plates were further incubated at 37 °C for 18 h; at the end of the incubation period, the MIC values of tested compounds were determined by visual inspection.

### 3.9. Antichlamydia Activity: Growth in Hela Cells, Cultivation and Quantification

The *Chlamydia trachomatis* strain was propagated and partially purified as described previously [60] with some modifications [61]. The infected cells were stained with monoclonal antichlamydia lipopolysaccharide (LPS) antibody (AbD Serotec, Oxford, UK) and fluorescein isothiocyanate-labeled anti-mouse IgG (Sigma-Aldrich) and the number of cells containing chlamydial inclusions was counted under a UV microscope, and the titer was expressed as the number of inclusion forming units (IFU)/mL.

One day before the infection and treatment the cells were HeLa cells were transferred into a 96-well plate at the same density as described vide supra. Next day for C. trachomatis infection, the wells were washed twice with 100 μL/well of PBS. The metal complexes were diluted in culture medium with cycloheximide. Concentration ranges of 0.048–100 μM for complexes **1**–**4**; **6** and RAPTA-C, 0.024–50 μM for complexes **7** and **8**, and 0.012–25 μM for complex **5** with 2-fold dilutions were tested. The cells were centrifuged for 1 h at 800× *g* for the infection with C. trachomatis at MOI 0.2. After the infection the cells were washed twice with PBS and the culture medium with cycloheximide was supplemented with the serial 2-fold dilutions of the respective solution and was added to triplicate wells. The plates were incubated for 48 h at 37 °C, 5% CO_2_. The plates were evaluated with quantitative PCR.

### 3.10. Antiviral Activity: Growth in Vero Cells, Cultivation and Quantification

Human herpes simplex virus-2 (HSV-2) strain was (gift from Dr. Ilona Mucsi, University of Szeged, Szeged, Hungary) grown in Vero (ATCC) cells and the infectivity was measured by using the plaque titration method [62]. The virus titer was expressed in plaque forming units (PFU) [63].

Vero cells (ATCC) were transferred into the wells of the 96-well plate at a density of 4 × 10^4^ cells/well in 100 μL of Dulbecco’s Modified Eagle’s Medium (DMEM, Sigma) containing 5% FBS, 0.14% sodium bicarbonate, 100 U/mL penicillin, 100 mg/mL streptomycin sulfate, and 0.25 g/mL amphotericin B. Before the infection the cells were washed twice with PBS, then the cells were incubated HSV-2 (MOI 0.2) for 1 h at 37 °C under a 5% CO_2_ atmosphere. After the infection the cells were washed twice with PBS again and the culture medium with cycloheximide was supplemented with the serial 2-fold dilutions of the complexes and was added to triplicate wells. Concentration ranges of 0.048–100 μM for complexes **1**–**4**; **7** and **8**, 0.024–50 μM for RAPTA-C, and 0.003–6.25 μM for complexes **5** and **6** with 2-fold dilutions were tested. The plates were incubated for at 37 °C, 5% CO_2_ for 24 h. The plates were evaluated with quantitative PCR.

### 3.11. Chlamydia Trachomatis and HSV-2 Growth Monitoring by Direct Quantitative PCR

At the end of the infection (24 h for HSV-2, 48 h for *C. trachomatis*) the supernatants of the cells were removed and the cells were washed with PBS twice, and finally 100 μL in sucrose-phosphate-glutamic acid buffer (SPG) was measured into the wells, and the cells were subjected to two freeze-thaw cycles. The mixed cell lysates were used as a template in the qPCR as described previously [64]. Briefly, qPCR was performed with the 5× HOT FIREPol^®^EvaGreen^®^qPCR Supermix (Solis Solis BioDyne, Tartu, Estonia) and *C. trachomatis pykF* gene specific primer pair in a Bio-Rad CFX96 real time PCR system. The *pykF* primer sequences were the following: 5′-GTTGCCAACGCCATTTACGATGGA-3′, 5′-TGCATGTACAGGATGGGCTCCTAA-3′. For each sample the cycle threshold (Ct) number corresponding to the cycle where the amplification curve crossed the base line was determined. For the quantitative measurement of HSV-2 growth a similar qPCR method was used as was applied for *C. trachomatis*. The HSV-2 *g*D2 gene specific primer pair (*g*D2-F: 5′-TCAGCGAGGATAACCTGGGA-3′, *g*D2-R 5′-GGGAGAGCGTA CTTGCAGGA-3′ was used with a 69 °C annealing-extension temperature [64,65].

## 4. Conclusions

All chloride complexes (**1**, **3**, **5**, **7**) as well as PTA complex **8** were active against Gram-positive *S. aureus* and *E. faecalis*, but were found to be ineffective on the tested Gram-negative strains. The coordination of the co-ligand PTA thus largely suppresses the antibacterial activity of these organoruthenium compounds. Chlorido complex **3** with *O*,*S*-ligand pyrithione exhibited the most remarkable antibacterial effect against the methicillin-resistant *S. aureus* (MRSA) strain. Complexes **1**–**8** and RAPTA-C displayed antichlamydia activity in human cervix carcinoma HeLa 229 cells. It was also found that among the tested compounds, complex **7** with *O*,*O*-ligand could inhibit the growth of the human herpes simplex virus-2. Chlorido complexes **1**, **3** and **5** exhibited the highest cytotoxicity among the tested compounds on Colo 205 and Colo 320 human adenocarcinoma cell lines, while the *β*-diketone complexes **7** and **8** displayed low activity most probably due to their lower solution stability. Therefore, we have clearly showed that some of our compounds possess potentially useful combinations of therapeutic effects (anticancer-antibacterial; anticancer-antiviral). Comparing the analogous chlorido and PTA complexes, it can be concluded that the coordination of PTA diminished the cytotoxic activity against the monitored cancer cells. In case of the pyridine-/isoquinolinethione complexes, the benzene condensation resulted in a more lipophilic, somewhat less cytotoxic but more selective compound. Based on the rhodamine 123 assay, the ABCB1-modulating ability of complexes **3** and **5** was highlighted.

Detailed speciation studies performed in aqueous solution revealed the high stability of pyrithione-type complexes **1**–**4** in a wide pH-range. Complexes containing PTA (**2**, **4**) were unchanged in the monitored pH range (2–11), while hydroxido complexes were formed in case of the chlorido compounds in the basic pH range. The process was characterized by high pK_a_ values (10.37 (**1**) and 10.29 (**3**)), therefore, hydroxido complexes are not present at physiological pH. Chlorido complexes can suffer arene loss at ligand excess or by the addition of phen, while the PTA containing complexes remain intact. The chlorido co-ligand can be partly exchanged with water in complexes **1** and **3**, and as a result the lipophilicity of the complexes is decreased with decreasing chloride ion concentration. As the chloride ion concentration is lower in the intracellular fluid than in the blood plasma, these complexes can undergo a higher extent of aquation when enter cells; thus, they may become activated upon the chlorido/water co-ligand exchange, similarly to the activation of cisplatin. The complexes showed good membrane permeability, which is increased by the coordination of PTA and the condensation of the benzene ring. 

Complexes **1**–**4** are able to interact with HSA, and the chlorido complexes displayed faster and stronger binding in comparison to the PTA-containing compounds. This binding preference was also reflected in their reaction with the monodentate binding model 1-methylimidazole. Binding of complexes **1** and **3** to HSA results in a significant Trp-quenching, and WF and DG displacement experiments also suggest the strong binding of these complexes at both sites I and II.

## Figures and Tables

**Table 1:** IC_50_ values of the complexes determined on chemo-sensitive (Colo 205), multidrug resistant (Colo 320) human colonic adenocarcinoma cell lines and normal human embryonal lung fibroblast cells (MRC-5) (72 h), in addition to selectivity indexes (S.I., ratio of the indicated IC_50_ values).

	IC_50_ (μM)			S.I.	
	Colo 205	Colo 320	MRC-5	MRC-5/Colo 205	MRC-5/Colo 320
**1**	14.04 ± 0.62	3.3 ± 1.3	2.17 ± 0.22	0.15	0.66
**2**	>100	>100	>100	–	–
**3**	17.3 ± 3.1	10.7 ±1.5	7.6 ± 1.6	0.44	0.71
**4**	>100	>100	81.0 ± 6.2	<0.81	<0.81
**5**	21.0 ± 2.4	13.74 ± 0.85	2.95 ± 0.71	0.14	0.21
**6**	>100	>100	>100	–	–
**7**	52.4 ± 2.7	29.1 ± 2.0	26.5 ± 3.2	0.51	0.91
**8**	80.6 ± 1.1	47.8 ± 7.3	17.6 ± 1.5	0.22	0.37
RAPTA-C	>100	>100	>100	–	–
cisplatin	29.8 ± 1.2	5.58 ± 0.70	0.88 ± 0.09	0.03	0.16

**Table 2:** Antibacterial activity of the complexes on Gram-positive and Gram-negative bacterial strains. MIC: minimum inhibitory concentration.

MIC (μM)	*S. aureus* ^1^	*E. faecalis* ^2^	*E. coli* ^3^	*K. pneumoniae* ^4^
	*Gram-Positive*		*Gram-Negative*	
**1**	50	100	>100	>100
**2**	>100	>100	>100	>100
**3**	12.5	12.5	>100	>100
**4**	>100	>100	>100	>100
**5**	25	12.5	>100	>100
**6**	100	>100	>100	>100
**7**	50	50	>100	>100
**8**	50	100	>100	>100
RAPTA-C	>100	>100	>100	>100
cisplatin	>100	>100	>100	>100

^1^ MRA ATCC 43300. ^2^ ATCC 29212. ^3^ AG100. ^4^ ATCC 49619.

**Table 3:** p*K*_a_ values of ligands H**PYR** and H**HiQT** and chlorido complexes **1** and **3** in pure water in the presence of 200 mM chloride ions. {*T* = 25.0 °C, *I* = 0.2 M KCl}.

Compound	p*K*_a_	Method	*c*
H**PYR**	4.52 ± 0.04 ^1^	pH-potentiometry	1.3 mM
H**HiQT**	4.63 ± 0.08	UV-vis	27 μM
**1**	10.37 ± 0.06	pH-potentiometry	1.30 mM
	10.34 ± 0.03	UV-vis	250 μM
**3**	10.29 ± 0.09	pH-potentiometry ^2^	0.6 mM
	10.25 ± 0.03	UV-vis	250 μM

^1^ pK_a_ = 4.49 (I = 0.20 M KCl) [40]. ^2^ Complex **3** has much better water solubility than its ligand H**iQT**.

**Table 4:** Log *D*_7.4_ (*n*-octanol/water) and effective passive permeability values (*P*_eff_) of complexes **1–3** at pH = 7.4. {*T* = 25.0 °C, 20 mM phosphate buffer}.

	c(KCl)	1	2	3
log*D*_7.4_	4 mM	−0.43 ± 0.09	n.d.	+0.92 ± 0.06
log*D*_7.4_	24 mM	−0.10 ± 0.05	n.d.	+1.22 ± 0.04
log*D*_7.4_	100 mM	+0.31 ± 0.03	n.d.	+1.37 ± 0.06
*P*_eff_ (cm/s)	100 mM	1.13 × 10^−6^	2.88 × 10^−6^	3.35 × 10^−6^
recovery		1.3%	15%	8.1%

**Table 5:** Conditional binding constants of the compounds at binding sites I and II of HSA determined by spectrofluorometric Trp-214 quenching (log*K*_Q_’) and site marker (WF or DG) displacement (log*K*_WF_’ and log*K*_DG_’) measurements. {pH = 7.40 PBS’ buffer; *T* = 25 °C}.

	log*K*_Q_’		log*K*_WF_’	log*K*_DG_’
**1**	5.81 ± 0.03	*(titration)*	6.16 ± 0.03	5.80 ± 0.03
**2**	<4	*(titration)*	–	–
**3**	6.18 ± 0.03	*(titration)*	5.98 ± 0.03	5.61 ± 0.03
**4**	4.46 ± 0.03 5.39 ± 0.03	*(titration)* (48 h)	–	–

## Data Availability

Authors can confirm that all relevant data are included in the article.

## Supplementary Materials

The following are available online at https://www.mdpi.com/article/10.3390/ph14060518/s1, Table S1: ABCB1 modulating activity on multidrug-resistant Colo 320 colonic adenocarcinoma cells in the presence of complexes 1–8, RAPTA-C. Scheme S1: Some selected equilibrium processes taking place in the solution of [Ru(η^6^-*p*-cymene)(X,Y)(Z)] complexes. Figure S1: Cytotoxicity of the complexes in HeLa cells. Figure S2: Antibacterial effect of complexes against *Chlamydia trachomatis*. Figure S3: Estimation of the direct impact of complexes and RAPTA-C on the qPCR, calculated dCt values of the *C. trachomatis* inhibition/stimulation test of the complexes and RAPTA-C. Figure S4: Cytotoxicity of the complexes in Vero cells. Figure S5: Antiviral effect of complexes 5, 6 and RAPTA-C. Figure S6: Time-dependent UV-vis spectra of 5 and 6 recorded in EMEM. Figure S7: Time-dependent UV-vis spectra of 7 and 8 recorded in PBS’ at pH 7.4. Figure S8: ^1^H NMR spectra of 8 at pH 7.4 recorded after 10, 24 and 48 h waiting time. Figure S9: ^1^H NMR spectra recorded for [Ru(η^6^-*p*-cymene)(H_2_O)_3_]^2+^ and for complex 1 after 24 h waiting time at pH 1. Figure S10: Molar absorbance values obtained at various concentrations of complex 1 and 3. Figure S11: Time-dependence of UV-vis absorption spectra recorded for complex 1 in the presence of 2 equivalents of phen at pH = 7.4. Figures S12 and S13: UV-vis absorption spectra recorded for complex 1 and 3 at various concentrations of PTA at pH = 7.4, respectively.
