# Supplementary material for: Comparison of Solution Chemical Properties and Biological Activity of Ruthenium Complexes of Selected β-Diketone, 8-Hydroxyquinoline and Pyrithione Ligands
Source: Pharmaceuticals (Basel). 2021 May 27;14(6):518. doi: 10.3390/ph14060518 (PMC8226722; doi:10.3390/ph14060518)
Supplement: Supplementary file 1 [file pharmaceuticals-14-00518-s001.zip › pharmaceuticals-1217001-supplementary.pdf]

## Supplementary Materials

# Comparison of solution chemical properties and biological activity of ruthenium complexes of selected *β*-diketone, 8-hydroxyquinoline and pyrithione ligands

Tamás Pivarcsik, Gábor Tóth, Nikoletta Szemerédi, Anita Bogdanov, Gabriella Spengler, Jakob Kljun, Jerneja Kladnik, Iztok Turel, Éva A. Enyedy

**Table S1.** ABCB1 modulating activity on multidrug-resistant Colo 320 colonic adenocarcinoma cells in the presence of complexes **1-8**, RAPTA-C at 2 μM and 20 μM concentrations. Verapamil (Verap.) was used as a positive control at 20 μM. The following fluorescence intensities were determined: forward scatter count (FSC), side scatter count (SSC) and mean fluorescence of the cells (FL-1) at 2 μM and 20 μM concentrations. FAR: fluorescence accumulation ratio, FAR quotient: FAR of compounds related to that of 20 μM verapamil.

| Sample | Conc. (μM) | FCS | SSC | FL-1 | FAR | FAR quotient (%) |
|---|---|---|---|---|---|---|
| **Verap.** | 20 | 1973 | 911 | 25.4 | 4.81 | 100 |
| **1** | 2 | 1949 | 868 | 6.18 | 1.17 | 24.3 |
| | 20 | 2068 | 978 | 5.53 | 1.05 | 21.8 |
| **2** | 2 | 2034 | 962 | 5.4 | 1.02 | 21.3 |
| | 20 | 2071 | 1024 | 5.41 | 1.02 | 21.3 |
| **3** | 2 | 2054 | 974 | 7.76 | 1.47 | 30.6 |
| | 20 | 1989 | 922 | 13.5 | 2.56 | 53.1 |
| **4** | 2 | 2014 | 959 | 4.33 | 0.82 | 17.0 |
| | 20 | 1977 | 973 | 8.95 | 1.70 | 35.2 |
| **5** | 2 | 1917 | 840 | 32.7 | 6.19 | 128.7 |
| | 20 | 1950 | 877 | 19.3 | 3.66 | 76.0 |
| **6** | 2 | 2003 | 965 | 4.52 | 0.86 | 17.8 |
| | 20 | 1912 | 865 | 3.68 | 0.70 | 14.5 |
| **7** | 2 | 1988 | 946 | 5.86 | 1.11 | 23.1 |
| | 20 | 1909 | 900 | 6.94 | 1.31 | 27.3 |
| **8** | 2 | 1925 | 877 | 3.51 | 0.66 | 13.8 |
| | 20 | 1950 | 917 | 5.68 | 1.08 | 22.4 |
| **RAPTA-C** | 2 | 1969 | 967 | 4.02 | 0.76 | 15.8 |
| | 20 | 2014 | 995 | 3.87 | 0.73 | 15.2 |
| **DMSO** | 2% | 1950 | 959 | 2.98 | 0.56 | 11.7 |

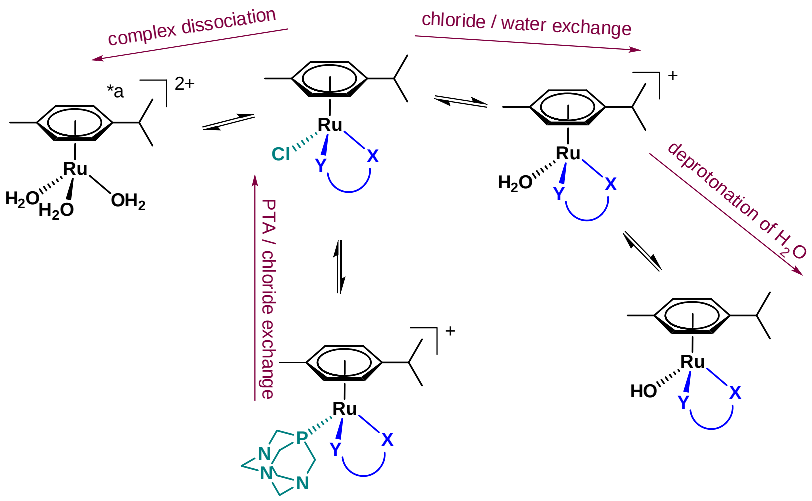


**Scheme S1.** Some selected equilibrium processes taking place in the solution of [Ru($\eta^6$-*p*-cymene)(X,Y)(Z)] complexes, where (X,Y) is a bidentate ligand, Z is the co-ligand $Cl^-$ or PTA. *a The actual chemical form of the ligand-free [Ru($\eta^6$-*p*-cymene)($H_2O$)$_3$]$^{2+}$ depends on the pH and the chloride ion content of the solution [27].

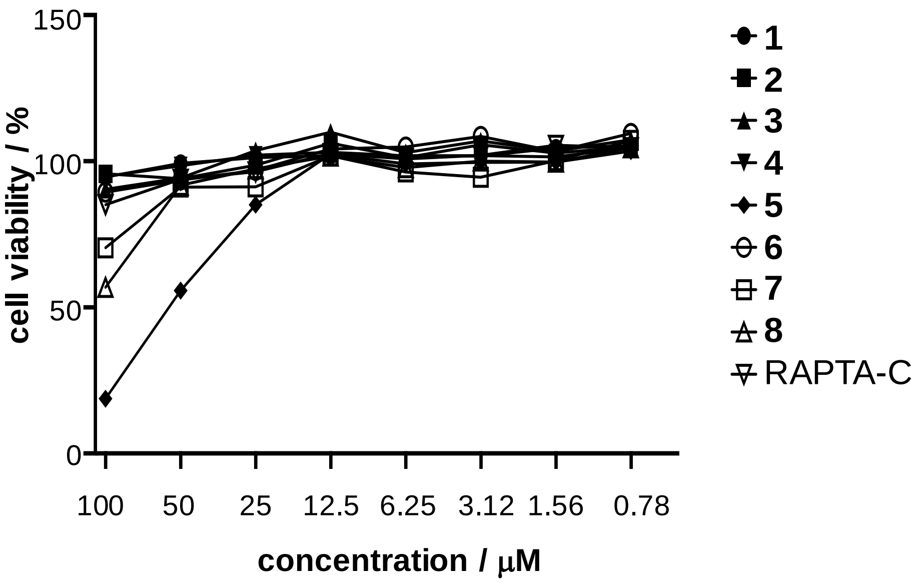


**Figure S1**. Cytotoxicity of the complexes in HeLa cells. {48 h} Complexes **1**–**4**, **6** and RAPTA-C did not produce significant toxicity.

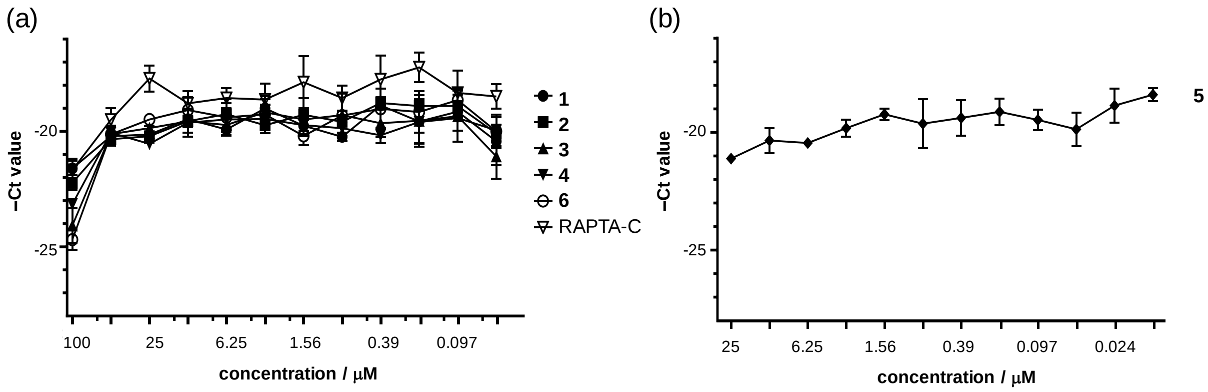


**Figure S2**. Antibacterial effect of complexes (**a**) **1**–**4**, **6** and RAPTA-C and (**b**) **5** against *Chlamydia trachomatis*.

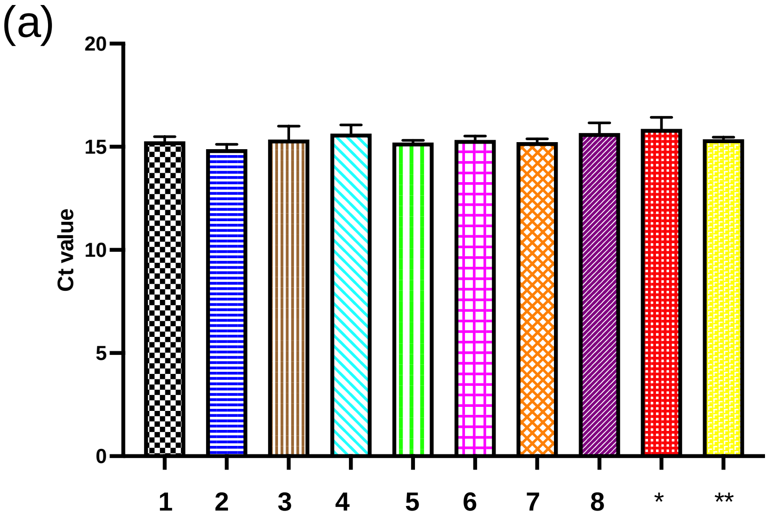


(b)

| complexes | dCt value |
|---|---|
| 1 | -0.093 |
| 2 | -0.480 |
| 3 | -0.013 |
| 4 | 0.284 |
| 5 | -0.156 |
| 6 | -0.033 |
| 7 | -0.140 |
| 8 | 0.304 |
| RAPTA-C | 0.501 |

**Figure S3**. (**a**) Estimation of the direct impact of complexes on the qPCR (*: RAPTA-C; **: untreated). Cell lysates of HeLa cells infected with untreated *C. trachomatis* (MOI 0.2, 48 h) mixed with cell lysates from uninfected HeLa cells treated with SPG solution contained 200 µM **1**–**4**, **6** and RAPTA-C complexes, 100 µM **7**, **8** complexes and 50 µM complex **5**, respectively. Ct values were compared to the mixture of untreated *C. trachomatis* infected cells and uninfected cells. Briefly, an enzyme stimulatory effect would evidence as a false chlamydial growth increasing effect, and the enzyme inhibitory effect during the qPCR would evidence as a false anti-chlamydia activity. If there was no direct impact of complexes on the qPCR, then the Ct level of the 1:1 mixture (basically a two-fold dilution of the chlamydial DNA) of the infected and uninfected but complexes containing cell lysates would have been similar to that of the *C. trachomatis* infected cells and uninfected lysate's. (**b**) The calculated dCt values of the *C. trachomatis* inhibition/stimulation test. The Ct levels of the *C. trachomatis* + complexes mixtures were under 1 dCt value than the untreated *C. trachomatis* infected cell lysate's.

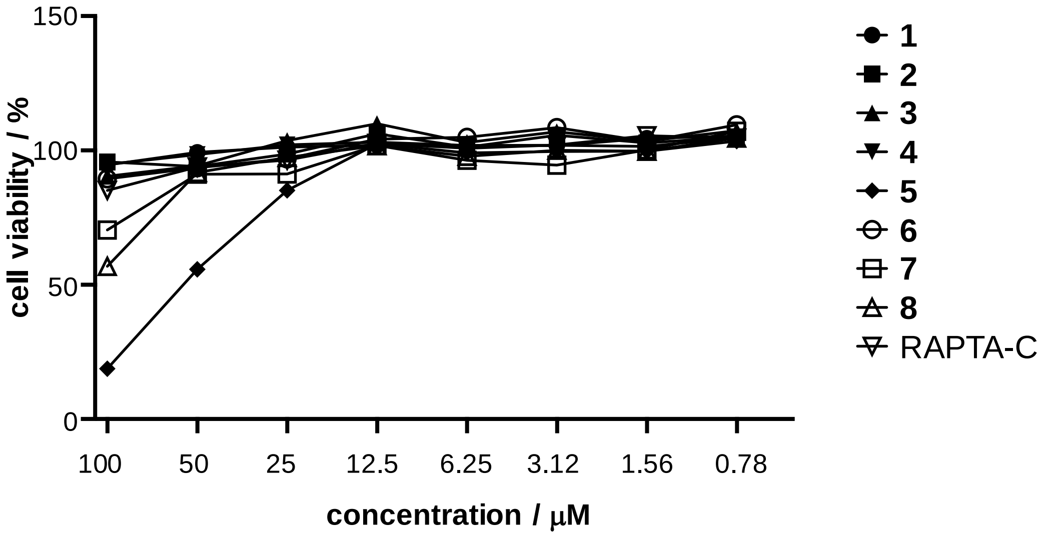


**Figure S4**. Cytotoxicity of the complexes in Vero cells. {24 h} Complexes **1**–**4**, **7** and **8** did not produce significant toxicity at any of the concentrations.

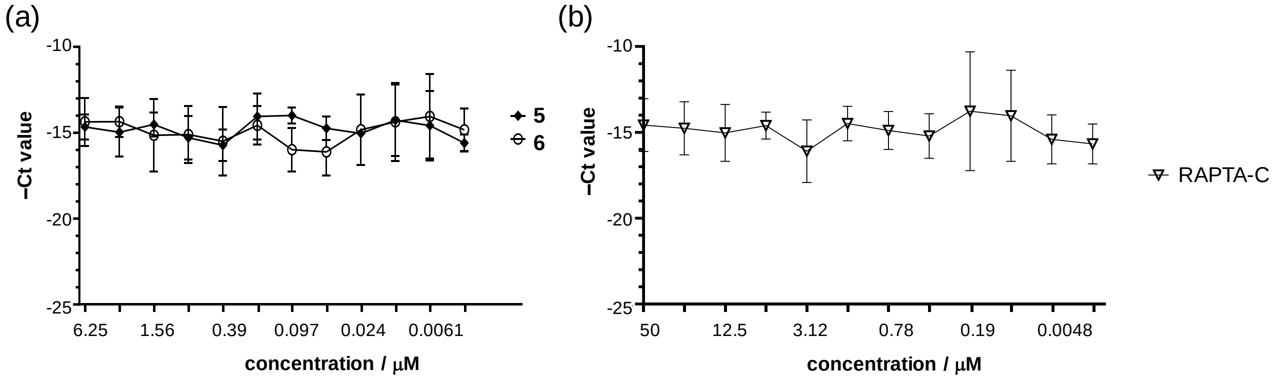


**Figure S5**. Antiviral effect of complexes (**a**) **5** and **6,** and (**b**) RAPTA-C against herpes simplex virus-2.

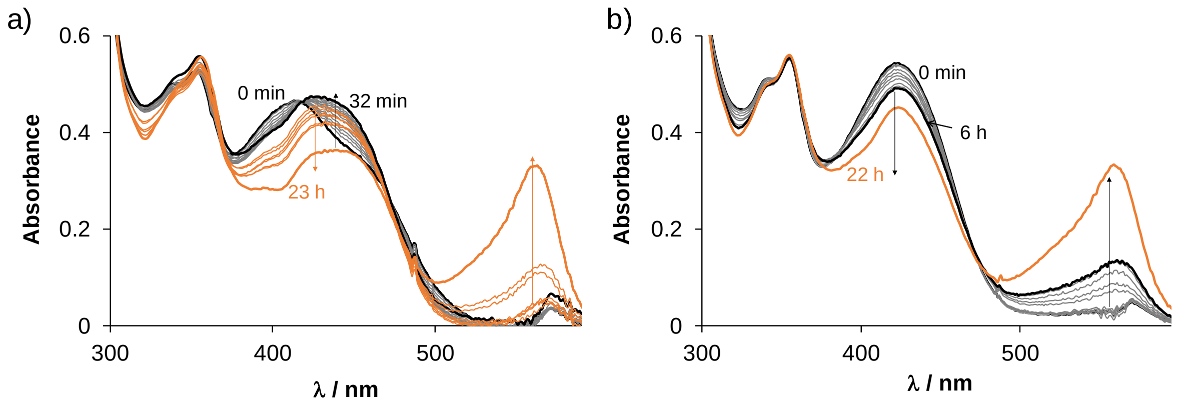


**Figure S6.** Time-dependent UV-vis spectra of (**a**) **5** and (**b**) **6** recorded in EMEM. {$T$ = 25.0 °C, $c_{complex}$ = 150 µM; 6% (v/v) DMSO; $l$ = 1 cm}

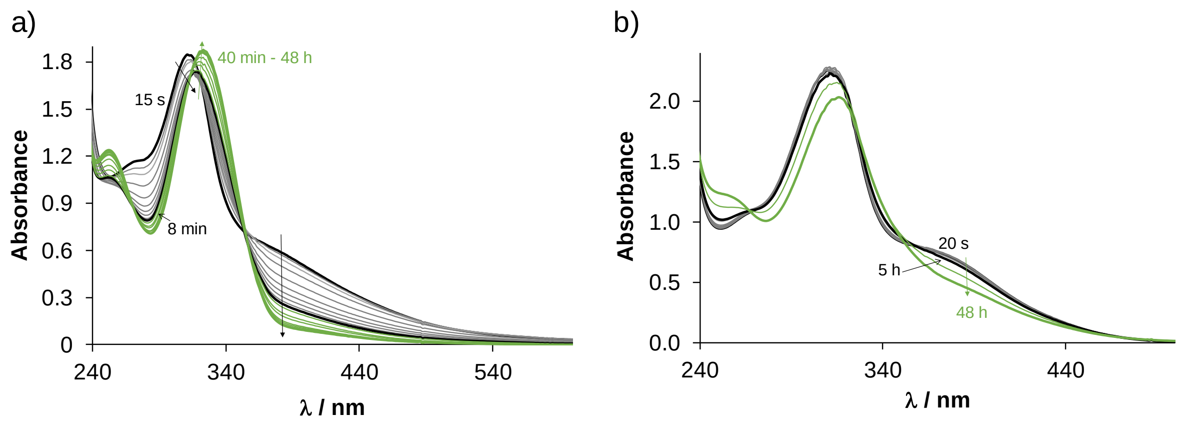


**Figure S7.** Time-dependent UV-vis spectra of (**a**) **7** and (**b**) **8** recorded in PBS' at pH 7.4. {$T$ = 25.0 °C, $c_{complex}$ = 160 μM; 2% (v/v) DMSO; $l$ = 1 cm (**7**) and 0.5 cm (**8**)}

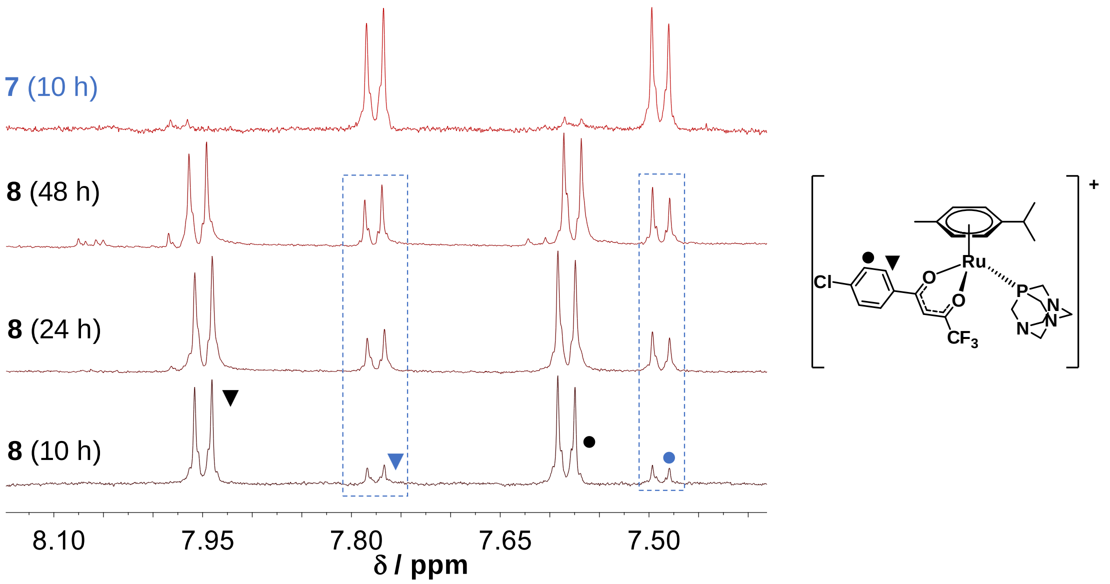


**Figure S8.** $^1$H NMR spectra of **8** at pH 7.4 (PBS') in the low field region recorded after 10, 24 and 48 h waiting time (and spectrum of **7** at 10 h for comparison). The blue frame indicates peaks belonging to formed chlorido complex **7**. {$T$ = 25.0 °C, $c_8$ = 0.5 mM; 10% (v/v) DMSO-d$_6$}

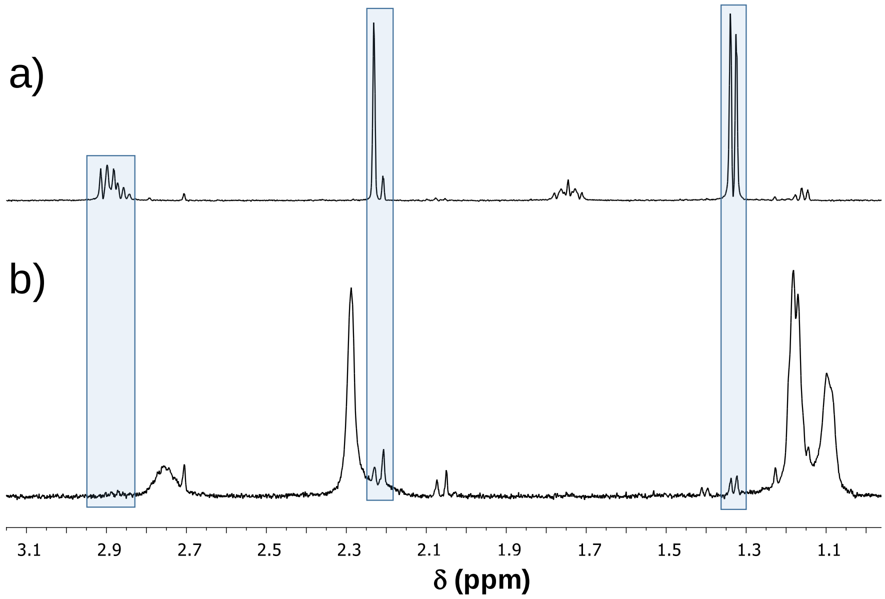


**Figure S9.** $^{1}$H NMR spectra in the high field region recorded for (**a**) [Ru($\eta^6$-*p*-cymene)($H_2O$)$_3$]$^{2+}$ and for (**b**) complex **1** after 24 h waiting time at pH 1 in the presence of 200 mM chloride ions. Frames peaks denote the unbound [Ru($\eta^6$-*p*-cymene)($H_2O$)$_3$]$^{2+}$. {$T$ = 25.0 °C, $I$ = 0.2 M KCl, $c_1$ = 1 mM; 10% (v/v) $D_2O$}

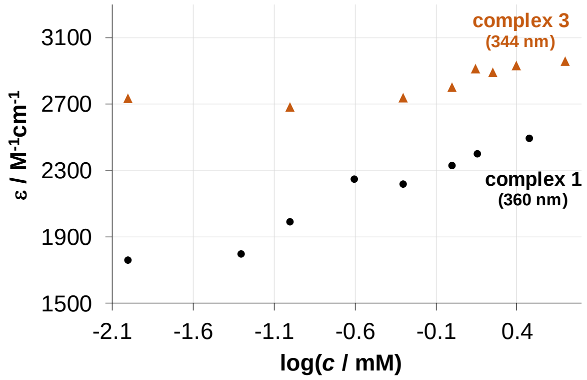


**Figure S10.** Molar absorbance ($\varepsilon$) values obtained at various concentrations of complex **1** (at 360 nm) and complex **3** (at 344 nm). {$T$ = 25.0 °C, $c_{complex}$ = 0.01–3 mM, path length = 1–50 mm, $I$ = 0.20 M KCl, pH = 7.4 (20 mM phosphate buffer)}

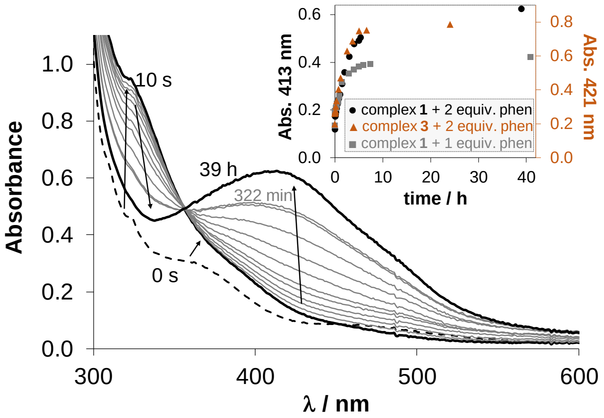


**Figure S11.** Time-dependence of UV-vis absorption spectra recorded for complex **1** in the presence of 2 equivalents of phen at pH = 7.4. Inserted figure shows the absorbance values plotted against the time for complex **1** with 1 equiv. phen (■), 2 equiv. phen (●) at 413 nm and for complex **3** with 2 equiv. phen (▲) at 421 nm. {$T$ = 25.0 °C, $c_{complex}$ = 100 µM, pH = 7.40 PBS' buffer}

*Explanation:* The use of a competitor ligand is often an advantage for stability constant determination, thus 1,10-phenantroline (phen) was added to the complexes **1** and **3** for this purpose. However, instead of a simple O,S-ligand/N,N-phen exchange process large and time-dependent spectral changes were observed, which were identified as the replacement of *p*-cymene, similarly as it was reported for Ru($\eta^6$-*p*-cymene)–8-hydroxyquinoline complexes [Mészáros, P. J.; Poljarevic, J. M.; Szatmári, I.; Csuvik, O.; Fülöp, F.; Szoboszlai, M.; Spengler, G.; Enyedy, É. A. An 8-hydroxyquinoline-proline hybrid with multidrug resistance reversal activity and solution chemistry of its half-sandwich organometallic Ru and Rh complexes. Dalton Trans. 2020, 49, 7977-7992]. Interestingly, the absorbance–time curves (see the inserted figure) have a biphasic character, which makes it probable that two reactions take place: first the fast formation of a Ru($\eta^6$-*p*-cymene)–ligand–phen mixed complex and then in a much slower reaction the *p*-cymene is released leading to the formation of a non-organometallic complex *e.g.* $[Ru(ligand)(phen)_2]^+$ at 2 equiv. phen. It should be noted that the detected arene loss is much slower than in case of the 8-hydroxyquinoline complexes. It was also found that addition of the O,S-ligand PYR or HiQT to the chlorido complexes **1** and **3**, respectively, also leads to the loss of the *p*-cymene ring. In contrast, addition of phen to the PTA containing complexes **2** and **4** does not result in arene loss.

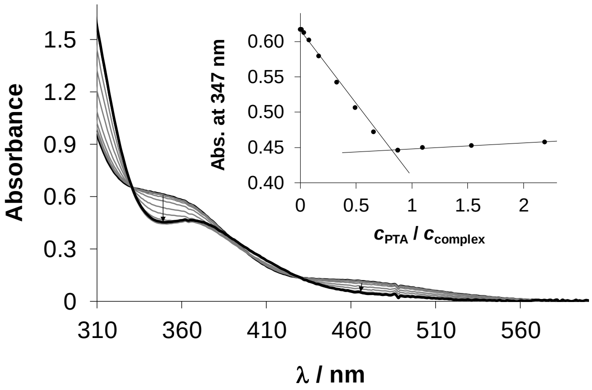


**Figure S12.** UV-vis absorption spectra recorded for complex **1** at various concentrations of PTA at pH = 7.4. Inserted figure shows the absorbance values at 347 nm plotted against the ratio of PTA and the complex. {$T$ = 25.0 °C, $c_{\text{complex } \mathbf{1}}$ = 193 μM, 2% (v/v) DMSO, 20 mM phosphate buffer; $l$ = 1 cm}

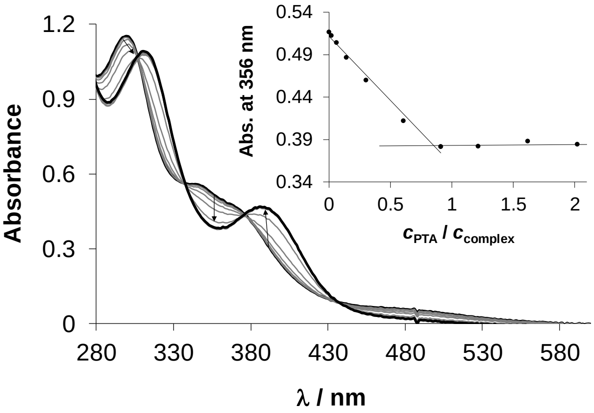


**Figure S13.** UV-vis absorption spectra recorded for complex **3** at various concentrations of PTA at pH = 7.4. Inserted figure shows the absorbance values at 356 nm plotted against the ratio of PTA and the complex. {$T$ = 25.0 °C, $c_{\text{complex } \mathbf{3}}$ = 104 μM, 2% (v/v) DMSO, 20 mM phosphate buffer; $l$ = 1 cm}
